# The Stem Species of Our Species: A Place for the Archaic Human Cranium from Ceprano, Italy

**DOI:** 10.1371/journal.pone.0018821

**Published:** 2011-04-20

**Authors:** Aurélien Mounier, Silvana Condemi, Giorgio Manzi

**Affiliations:** 1 Unité Mixte de Recherche 6578 – Unité d'Anthropologie Bioculturelle, Centre National de la Recherche Scientifique/Université de la Méditerranée/Etablissement Français du Sang, Marseille, France; 2 Dipartimento di Biologia Ambientale, Sapienza - Universitá di Roma, Roma, Italia; 3 Istituto Italiano di Paleontologia Umana, Roma, Italia; State University of New York College at Oneonta, United States of America

## Abstract

One of the present challenges in the study of human evolution is to recognize the hominin taxon that was ancestral to *Homo sapiens*. Some researchers regard *H. heidelbergensis* as the stem species involved in the evolutionary divergence leading to the emergence of *H. sapiens* in Africa, and to the evolution of the Neandertals in Europe. Nevertheless, the diagnosis and hypodigm of *H. heidelbergensis* still remain to be clarified. Here we evaluate the morphology of the incomplete cranium (calvarium) known as Ceprano whose age has been recently revised to the mid of the Middle Pleistocene, so as to test whether this specimen may be included in *H. heidelbergensis*. The analyses were performed according to a phenetic routine including geometric morphometrics and the evaluation of diagnostic discrete traits. The results strongly support the uniqueness of *H. heidelbergensis* on a wide geographical horizon, including both Eurasia and Africa. In this framework, the Ceprano calvarium – with its peculiar combination of archaic and derived traits – may represent, better than other penecontemporaneous specimens, an appropriate ancestral stock of this species, preceding the appearance of regional autapomorphic features.

## Introduction

Human fossil discoveries, such as those of the Sierra de Atapuerca, Spain [1], [2], have revealed the evidence for the presence of hominins in Europe well before 500 ka, in contradiction with the so-called “short chronology” model [3].

For more than a decade, the adult calvarium found near Ceprano in southern Latium, Italy [4] supported this view, particularly in account of its archaic morphology [4], [5], its possible relationship with Oldowan Palaeolithic assemblages from the same area [6], and its supposed dating to 800-900 ka [4], [7]. Recently, however, the site has been re-dated on the basis of multidisciplinary investigations to the mid of the Middle Pleistocene, between 430 and 385 ka [8], [9]. By contrast, the peculiar morphology of the Ceprano calvarium has no equivalent in Europe or elsewhere and its taxonomic status has been so far controversial, being alternatively viewed as a “late” *H. erectus* [4], [10], a possible adult individual of *H. antecessor* [5], or the holotype of a new species named *H. cepranensis* [11]. A number of studies [5], [12]–[14] also highlighted phenetic links with Mid-Pleistocene fossils from Africa (e.g. Kabwe 1) and Europe (e.g. Petralona), identifying Ceprano as the possible representative of an ancestral stock of *H. heidelbergensis* [15]. At the same time, despite the diagnosis and the hypodigm of *H. heidelbergensis* are still to be clarified [16]-[19], this species could represent the taxon of origin of the divergence between two distinct lineages of the Middle Pleistocene [20], [21]: those of the Neanderthals in Europe and *H. sapiens* in Africa.

We argue that the morphology of Ceprano, in view of the new proposed chronology, may help to better evaluate the significance of *H. heidelbergensis* for human evolution [21]. Thus, our aim here is to reconsider Ceprano in a wide comparative framework. An original phenetic routine [18], [19] (see Methods) – based on both geometric morphometrics and the scoring of morphological features – was performed to evaluate the taxonomic status of this specimen with respect to samples grouped as Early Pleistocene fossils (*H. ergaster* and/or *H. erectus*), Mid-Pleistocene hominins usually referred to *H. heidelbergensis*, Neandertals, and anatomically modern humans. Overall, Ceprano has been compared to 42 fossils from Africa and Eurasia ranging from ∼1.8 Ma to ∼12 ka (Table 1) and to 68 Holocene modern humans (see, Table S1). A comparison of the Ceprano calvarium with such an extensive sample, especially considering the *H. neanderthalensis* and *H. sapiens* specimens, was never carried out before [5], [14].

**Table 1:** Specimens included in the analytical protocol.

Specimens	Chronology	Site	Analyses	Labels Figure 2
*Early Pleistocene*				
**D2280**	1.81±0.05 Ma	Dmanisi, Georgia	HC p, GM	**1**
**D2700**	1.81±0.05 Ma	Dmanisi, Georgia	HC p, GM	**2**
KNM-ER 1470	∼1.8 Ma	East Turkana, Kenya	GM	**3**
KNM-ER1813	1.86±0.08 Ma	East Turkana, Kenya.	HC g+p, GM	**4**
KNM-ER 3733	∼1.6 Ma	East Turkana, Kenya	HC g+p, GM	**5**
KNM-ER 3883	∼1.6 Ma	East Turkana, Kenya	HC g+p, GM	**6**
OH9	>1.47 Ma	Olduvai Gorges, Tanzania	HC p	**-**
BOU-VP-2/66	∼1.0 Ma	Bouri, Ethiopia	HC p	**-**
Sangiran 17	1-1.5 Ma	Java, Indonesia	HC p, GM	**7**
*Middle Pleistocene*				
**Ceprano**	900-450 ka	Ceprano, Italy	HC p, GM	**-**
SH5	350-530 ka	Sima de los Huesos, Atapuerca, Spain	HC g+p, GM	**-**
**Petralona**	150-250 ka	Petralona, Greece	HC g+p, GM	**-**
**Steinheim**	250 ka	Steinheim, Germany	HC g+p, GM	**-**
**Ehringsdorf H**	230 ka	Ehringsdorf, Germany	GM	**-**
**Irhoud 1**	130-190 ka	Jebel Irhoud, Morocco	HC g+p, GM	**-**
**Kabwe 1**	>125 ka	Kabwe, Zambia	HC g+p, GM	**-**
LH 18	129-108 ka	Laetoli, Tanzania	HC g+p, GM	**-**
Omo II	∼130 ka	Omo Kibish, Ethiopia	HC p+g	**-**
**Singa**	133±2 ka	Singa, Soudan	HC g+p, GM	**-**
ZH Skull III (ZH III)	400-780 ka	Zhoukoudian, China	HC g+p, GM	**-**
ZH Skull XII (ZH XII)	400-780 ka	Zhoukoudian, China	HC p, GM	**-**
Hexian	412±25 ka	Hexian, China	GM	
Dali	260-300 ka	Dali, China	HC g+p, GM	**-**
Jinniu Shan	200 ka	Jinniu Shan, China	HC g+p, GM	**-**
*Late Pleistocene*				
Ngandong 6 (Ng 6)	40-200 ka	Java, Indonesia	HC g+p, GM	**-**
Ngandong 14 (Ng 14)	40-200 ka	Java, Indonesia	HC g+p, GM	**-**
Ngawi 1	∼40 ka	Java, Indonesia	HC p, GM	**-**
**Saccopastore 1**	100-130 ka	Saccopastore, Italy	GM	**8**
**Gibraltar 1**	45-70 ka	Forbe's Quarry, Gibraltar	HC p, GM	**-**
**La Ferrassie1**	53-66 ka	La Ferrassie, France	HC g+p, GM	**9**
**La Quina H5**	∼65 ka	La Quina, France	HC p	**-**
**La Chapelle-aux-Saints**	∼50 ka	La Chapelle-aux-Saints, France	HC g+p, GM	**10**
**Guattari 1**	52±12 ka	Monte-Circeo, Italy	HC g+p, GM	**11**
**Spy 1**	>36 ka	Spy, Belgium	HC g+p, GM	**12**
**Tabūn I**	122±16 ka	Tabūn, Israel	GM	**13**
**Amud 1**	50-60 ka	Amud, Israel	GM	**14**
**Cro-Magnon I**	28 ka	Les Eyzies, France	HC g+p	**-**
**Abri Pataud 1**	22 ka	Les Eyzies, France	HC g+p, GM	**15**
Chancelade	∼12 ka	Chancelade, France	HC g+p, GM	**16**
**Qafzeh 9**	100-130 ka	Qafzeh, Israel	HC p, GM	**17**
**Qafzeh 6**	90-130 ka	Qafzeh, Israel	GM	**18**
**Skhūl V**	66-102 ka	Skhūl, Israel	HC g+p, GM	**19**
**Ohalo II**	19 ka	Ohalo, Israel	HC g+p, GM	**20**
*Holocene*				
**Hassi-el-Abiod** (N = 6)	6970±130 bp	Sahara, Mali	HC g+p, GM	-
**Loisy-en-Brie** (N = 12)	3740±120 bp	Loisy-en-Brie, France	HC g+p, GM	-
**Spitalfields** (N = 10)	XVII-XIX centuries	London, United Kingdom	HC g+p, GM	-
**Romania** (N = 10)	XIX century	Romania	HC g+p, GM	-
**China** (N = 10)	XX century	Chine – Tibet	HC g+p, GM	-
**Java** (N = 10)	XX century	Java – Maduras	HC g+p, GM	-
**Nigeria** (N = 10)	XX century	Nigeria	HC g+p, GM	-

Bold types indicate when original fossil was examined. Column “Analyses” indicates in which analyses the specimens were included: HC: hierarchical classification, g: general analysis, p: partial analysis; GM geometric morphometrics.

## Results

### Geometric morphometrics (shape analysis)

The M Box test results (M = 68.660, F = 0.945, ddl1 = 42, ddl2 = 860.99, p = 0.572) indicates that the covariance matrices are homogenous, and therefore a linear Discriminant Function Analysis (DFA) is appropriate.

The first discriminant function (F1) of the DFA accounts for 80.9% of the total variance; it clearly separates the three predefined groups (i.e. modern humans, Neandertals and Early Pleistocene fossils). The second function (F2: 19.9% of variance) more distinctly isolates the Neandertals. Wilks' lambda is significant (F1: Wilk's λ = 0.030, chi-square = 103.894, df = 12, *p*<0.0001; F2: Wilk's λ = 0.309, chi-square = 34.663, df = 5, *p*<0.0001) along both the first and second functions suggests that the variable can be used to distinguish between the groups.

Linear regressions (PC1: R^2^ = 0.013, *p* = 0.419; PC2: R^2^ = 0.041, *p* = 0.151; PC3: R^2^ = 0.001, *p* = 0.891; PC4: R^2^ = 0.055; *p* = 0.094; PC5: R^2^ = 0.057, *p* = 0.089; PC6: R^2^ = 0.008, *p* = 0.525) indicate that centroid size does not significantly impact specimens' shape (see, Table S8). Thus, differences in shape between specimens are not due to allometry.

**Figure 1:**
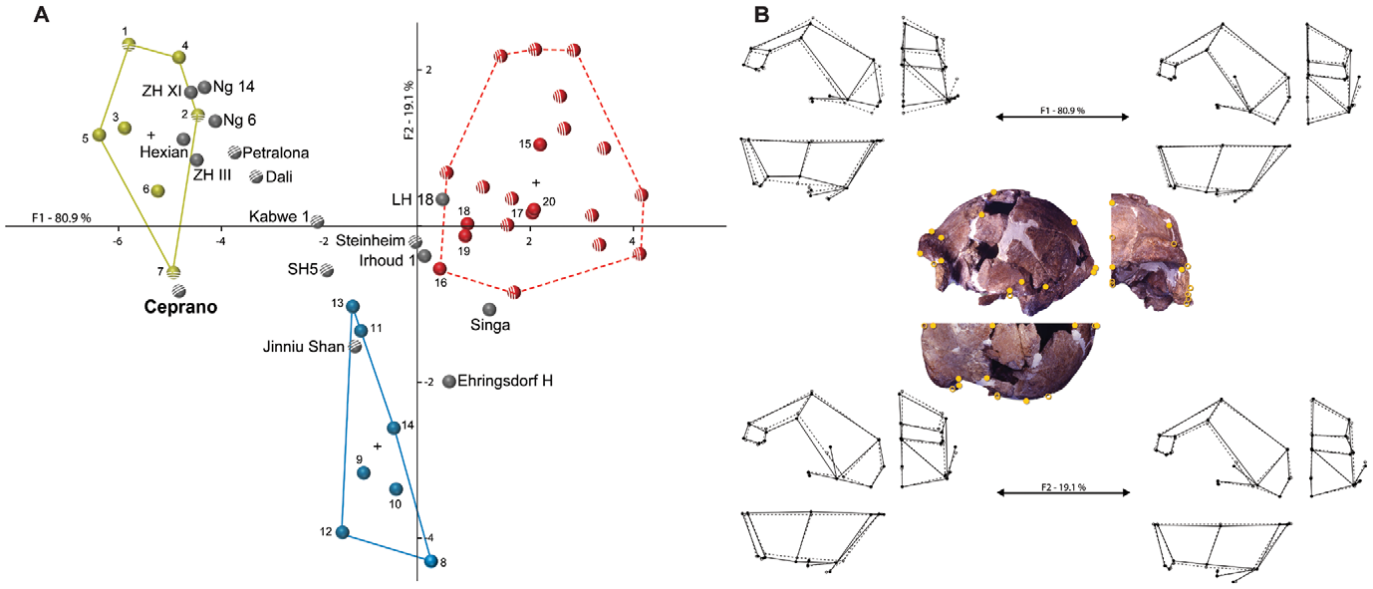
Discriminant Function Analysis based on landmarks data (A) and associated cranial shapes (B). A) Crosses indicate centroïds of each a priori sample. Red spheres  =  modern humans (plain, fossil specimens; stripe, Holocene specimens); blue spheres  =  Neandertals; green spheres  =  Early Pleistocene composite sample (plain, African specimens; stripe, Eurasian specimens); gray spheres  =  fossils included a posteriori in the analysis (stripe spheres are specimens that cluster with Ceprano in the dendrograms of Figure 2). B) The configuration of landmarks is indicated by yellow circles superimposed on views of the Ceprano cranium (full, visible landmarks; empty, landmarks non visible in the current view); shapes in norma lateralis (upper left), norma verticalis (lower left) and norma facialis (right) are portrayed for the extremities of each axis (full lines, shape change; dashed lines, consensus). Modern humans, Neandertals and Early Pleistocene specimens are well-discriminated on Function 1. Function 2 discriminates modern humans and Early Pleistocene specimens from Neandertals. The architectural shape of Ceprano is closer to Early Pleistocene specimens and, particularly, to Sangiran 17 from Java (**7**). Due to the apparent deformation of both Steinheim and Jinniushan, their respective positions in the graph are at least questionable.

The F1 is responsible for most of the dispersion of the cloud of points (Figure 1A). Neandertals and modern humans groups slightly overlap at the centre of the chart while Early Pleistocene specimens are better segregated. It is due to the large dispersion of the modern human group on F1. *H. sapiens* fossil specimens tend to be positioned well within or at the lower left margin of the recent human cloud of points, while Qafzeh 6, Skhūl V and Chancelade show similarities with the Neandertal shape. Saccopastore 1 shows extremes values compare to other Neandertals for both functions, while Tabun I and Guattari I are separated from the other Neandertals on F1. Early Pleistocene specimens are quite homogeneous on F1. The African and Dmanisi specimens show strong similarities in their shape, while Sangiran 17 is more isolated especially on F2.

We can elaborate on the distributions of calvaria shape among hominins if we look at Figure 1B. Extreme shapes for modern humans show an expansion of the calvaria in all dimensions: the vault is higher, wider and slightly longer. The supra-orbital region appears to display a weak projection and the *linea temporalis* insertions on the frontal and parietal are relatively lower. The Neandertals are characterized by a cranial vault slightly lower (bregma, landmark #4) and lengthen (opisthocranion, #2). Post-orbital region is concave: projecting in its medial part (nasion, #6) and is retreating laterally (fronto-malare orbital, #7). There is almost no post-orbital constriction. The occipital and parietal are more developed (asterion, #12) with a medially positioned euryon on the parietal which results in the characteristic “en bombe” form of the Neandertal calvaria in *norma occipitalis*. Early Pleistocene fossils show a lower calvaria, an almost straight post-orbital region with strong lateral and forward developments (nasion #6, and fronto-malare orbital #7). There is a strong post-orbital constriction and the insertions of the *linea temporalis* are in a high position on the frontal and parietal. The parietal is weakly developed notably due to the forward and high position of the lambda (#3), and the *planum occipitale* is well-developed with almost coincident inion (#1) and opisthocranion (#2).

The position on the scatter plot of other specimens (i.e. Ceprano, Mid-Pleistocene fossils and late *H. erectus*) has been calculated *a posteriori*. Ceprano does not present particular affinities with Mid-Pleistocene specimens and is most similar to Early Pleistocene specimens, especially to Sangiran 17. Ngandong fossils too show strong similarities with Early Pleistocene fossils, as it is the case for the shape of Asian fossils from Zhoukoudian and Hexian. Also, late Mid-Pleistocene fossils show important similarities with modern humans for the African (LH 18, Jebel Irhoud 1 and Singa) and with Neandertals for the European (Ehringsdorf H). Surprisingly, Steinheim seems to display similarities with modern humans, and Jinniu Shan with Neandertals. Finally, Petralona and Dali are similar to each other and are quite similar to Early Pleistocene specimens. This is also the case for Kabwe 1 although its shape is more transitional between Early Pleistocene specimens and modern humans. Kabwe 1 shares similarities with Dali and SH5, which in turn resembles much to Neandertals (Figure 1).

### Morphology (scored morphological features)

**Figure 2:**
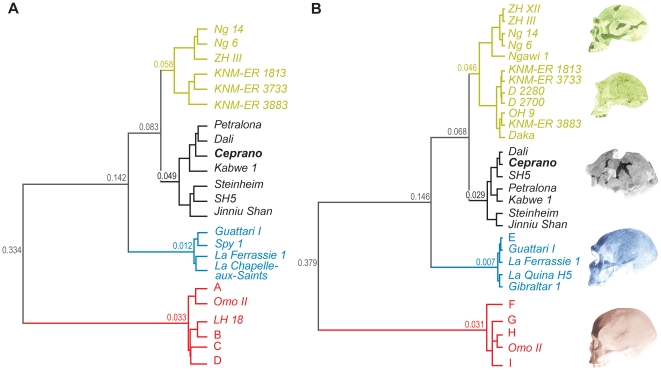
Hierarchical classification based on discrete features: general (A) and partial (B) analyses. Branches and number at nodes express morphological distance between clusters. Numbers represent groups of specimens that are displayed as clusters (**A**: Irhoud 1, Skhūl V; **B**: 4 Holocene specimens; **C**: 13 Holocene specimens; **D**: Singa, Cro-Magnon I, Abri Pataud 1, Chancelade, Ohalo II, 35 Holocene specimens; **E**: Spy 1, La Chapelle-aux-Saints; **F**: 14 Holocene specimens; **G**: Singa, LH 18, Cro-Magnon I, Ohalo II, 18 Holocene specimens; **H**: Irhoud 1, Skhūl V; **I**: Qafzeh 9, Abri Pataud 1, Chancelade, 35 Holocene specimens, see Figure S3 for details). Each cluster is described by statistically significant morphological features: pertinence criterion: T-Values>2 at *p*<0.05 (Tables 2, 3 and Table S15). The Partial analysis (B) allowed the inclusion of 8 additional specimens (Qafzeh 9, Gibraltar 1, La Quina H5, Ngawi 1, OH9, D2280, D2700 and Daka); 13 morphological features not preserved on these specimens (2, 10, 14, 15, 16, 18, 25, 26, 40, 41, 43, 47, 50) are not used in this analysis. In both analyses, Early Pleistocene specimens and *H. erectus* sensu stricto (China and Java) are distinguished as two sub-groups of the same cluster; modern humans are separated from all the other samples, but close to African late Mid-Pleistocene specimens such as Jebel Irhoud 1, Omo II, LH18, and Singa; Ceprano is always part of a the Mid-Pleistocene cluster, with African and Eurasian fossils.

The dendrogram from the global analysis (50 morphological features, Figure 2A, for more details see Figure S3A) displays a clear separation between the cluster which includes al modern humans and the other specimens based on chi-square dissimilarity index and which indicates a high degree of dissimilarity (dissimilarity value  = 0.334). The second node clearly segregates Neandertals from other Middle and Early Pleistocene specimens, as well as from Ngandong fossils (dissimilarity value  = 0.142) (Figure 2A). The next most discriminating node distinguishes Mid-Pleistocene specimens from Early Pleistocene and Asiatic *H. erectus* specimens (dissimilarity value  = 0.083). African fossils from late Middle Pleistocene (Omo II and Jebel Irhoud 1) and Skhūl V are part of the main modern humans cluster. The Ceprano cranium clusters with Mid-Pleistocene specimens. This group highest value of dissimilarity is 0.049 which corresponds to the separation of Jinniu Shan, SH5 and Steinheim from Kabwe 1, Ceprano, Dali and Petralona. Ceprano shows a strong association with Petralona and Dali (dissimilarity value 0.020) while Dali and Petralona present the lowest dissimilarity value of the cluster (0.015). Finally African Early Pleistocene specimens (i.e., KNM-ER 3733, KNM-ER 3883 and KNM-ER 1813) are separated from Asian *H. erectus* (i.e., ZH III, Ng 6 and 14) (Figure 2A).

39 morphological features can be identified as statistically significant (*p*<0.05) for the description of the modern humans cluster while 19 morphological features are associated with the Neandertal cluster. Neandertal and modern humans clusters are described by well recognize morphological features which are often used to define these two species (e.g. absence of torus occipitalis transversus, well-developed tuber parietale and tuber frontale, auditory meatus positioned under the processus zygomaticus temporalis for modern humans; presence of an occipital bun, presence of a suprainiac fossa, auditory meatus aligned with the processus zygomaticus temporalis for Neandertals; see Table S15). The Ceprano's cluster is characterized by 23 statistically significant morphological features (e.g. incomplete sulcus supraorbitalis, medially concave supra-orbital region in *norma verticalis*, medially shifted tuber frontale, presence of a torus angularis parietalis, intermediate position of the auditory meatus with regard to the processus zygomaticus temporalis), while 31 morphological features describe the Early Pleistocene and Asiatic *H. erectus* cluster (Table 2). As we emphasized, Ceprano shows strong similarities with Mid-Pleistocene specimens. Therefore, we may offer the inclusion of Ceprano among a possible taxon which will include a regrouping of African and European Mid-Pleistocene fossil specimens as well as more recent Asiatic specimens (i.e., Dali and Jinniu Shan).

**Table 2:** Hierarchical classification, general analysis (Figure 2A): description of Mid-Pleistocene and Early Pleistocene/Asians *H. erectus* clusters by the most relevant morphological features and character states.

Mid-Pleistocene				Early Pleistocene/Asians *H. erectus*			
Morphological features	character states	T-Values	*p*<	Morphological features	character states	T-Values	*p*<
Supra-orbital region: sulcus supraorbitalis	2	5.25	0.001	Bregmatic eminence	2	5.18	0.001
Sulcus postorbitalis	3	4.42	0.001	Outline of the superior border of the squama	2	4.84	0.001
Tuber frontale	2	4.33	0.001	Outline of the calvaria, norma lateralis	1	4.73	0.001
Torus occipitalis transversus	2	4.33	0.001	Tuber parietale	1	4.73	0.001
Projection of the supra-orbital region	3	4.33	0.001	Petro-tympanic crest orientation	3	4.73	0.001
Position of the auditory meatus	2	4.23	0.001	Torus occipitalis transversus	2	4.73	0.001
Outline of the supra-orbital region. norma verticalis	1	3.75	0.001	Outline of the supra-orbital region. norma verticalis	2	4.59	0.001
Sharply angulated occipital. norma lateralis	2	3.54	0.001	Opisthocranion coincident with inion	1	4.38	0.001
Petro-tympanic crest orientation	3	3.47	0.001	Sulcus supratoralis	3	4.06	0.001
Torus occipitalis transversus form in norma occipitalis	2	3.34	0.001	Sharply angulated occipital. norma lateralis	2	3.99	0.001
Tuberculum supramastoideum anterius	2	3.22	0.001	Sulcus postorbitalis	3	3.99	0.001
Linea temporalis forming a crest on the frontal	3	3.09	0.001	Projection of the supra-orbital region	3	3.90	0.001
Sagittal keel on the frontal	2	3.02	0.001	Tuber frontale	2	3.77	0.001
Occipital bun	2	3.02	0.001	Postorbital constriction	1	3.62	0.001
Torus angularis parietalis	2	3.02	0.001	Crista supramastoidea continues with the processus zygomaticus temporalis	2	3.31	0.001
Sulcus supratoralis	2	2.87	0.002	Sagittal keel on the frontal	2	3.26	0.001
Crista supramastoidea continues with the processus zygomaticus temporalis	2	2.80	0.003	Torus angularis parietalis	2	3.26	0.001
Processus retromastoideus	2	2.73	0.003	Frontal cord length/parietal cord length	3	3.06	0.001
Suprainiac fossa	2	2.59	0.005	Crista occipitomastoidea	2	2.98	0.001
Articular tubercle configuration	1	2.53	0.006	Juxtamastoid ridge development/processus mastoidus	2	2.98	0.001
Frontal cord length/parietal cord length	3	2.52	0.006	Processus retromastoideus	2	2.98	0.001
Tuberculum zygomaticum posterius	2	2.45	0.007	Supra-orbital region: sulcus supraorbitalis	2	2.86	0.002
Outline of the planum occipitalis. norma occipitalis	1	2.41	0.008	Development of the crista supramastoidea at the porion	3	2.86	0.002
-	-	-	-	Torus occipitalis transversus form in norma occipitalis	2	2.75	0.003
-	-	-	-	Articular tubercle configuration	3	2.70	0.003
-	-	-	-	Tuberculum supramastoideum anterius	2	2.64	0.004
-	-	-	-	Outline of the anterior border of the squama	2	2.60	0.005
-	-	-	-	Supramastoid groove	2	2.55	0.005
-	-	-	-	Sagittal keel on the bregma-lambda arc	2	2.36	0.009
-	-	-	-	Antero-posterior convexity of the frontal	1	2.33	0.010
-	-	-	-	Linea temporalis: superior line position on parietal	1	2.33	0.010

The statistical analysis identifies the character states that contribute the most to the formation of each class. The T-Value (pertinence criterion) must be ≥2 at *p*<0.05.

The partial analysis (Figure 2B, for more details see Figure S3B) was undertaken following the exclusion of 13 variables (see, Methods) that are not preserved or not available for Early Pleistocene specimens (D2280, D2700, Daka, OH9). First, there are no major changes concerning the Neandertals and modern humans clusters (Figure 2B). As in the general analysis, African specimens from the late Middle Pleistocene (Omo II, LH18, Singa and Jebel Irhoud 1) are part of the main modern humans cluster (Figure 2B). The Ceprano calvarium shows strong similarities with Mid-Pleistocene specimens, especially with the Dali cranium, but also with the fossil from Sima de los Huesos (i.e. SH5). The highest dissimilarity value for this group is lower than in the general analysis (0.030). There is little change concerning the set of morphological features that is significant for the description of the Ceprano cluster: the presence of a weakly-delineated suprainiac fossa and of a processus retromatsoideus are substituted by the presence of medially positioned tuber parietale (Table 3). Asian specimens referred to *H. erectus* (Zhoukoudian, Ngandong and Ngawi specimens) are discriminated from all Early Pleistocene African specimens. This cluster also includes the Georgian fossils from Dmanisi as well as the Daka cranium, which shares similarities with OH9 and KNM-ER 3883. Compared to the general analysis, the presence of a processus retromatoideus and a straight anterior border of the temporal squama are absent from the list describing this cluster. On the other hand, the set of morphological features includes a low temporal squama and a straight supra-orbital region in *norma facialis* as well as two character states (i.e. complete sulcus supraorbitalis and convex torus occipitalis transversus) which underline a relative morphological heterogeneity among the cluster. Indeed, other character states for these morphological features also describe the cluster (Table 3).

**Table 3:** Hierarchical classification. partial analysis (Figure 2B): description of Mid-Pleistocene and Early Pleistocene/Asians *H. erectus* clusters by the most relevant morphological features and character states.

Middle Pleistocene				Early Pleistocene – Asians *H. erectus*			
Morphological features	character states	T-Values	*p*<	Morphological features	character states	T-Values	*p*<
Supra-orbital region: sulcus supraorbitalis	2	5.08	0.001	Outline of the calvaria. norma lateralis	1	6.29	0.001
Tuber frontale	2	4.45	0.001	Outline of the superior border of the squama	2	6.24	0.001
Sulcus postorbitalis	3	4.36	0.001	Opisthocranion coincident with inion	1	6.13	0.001
Projection of the supra-orbital region	3	4.28	0.001	Petro-tympanic crest orientation	3	5.79	0.001
Position of the auditory meatus	2	4.16	0.001	Torus occipitalis transversus	2	5.79	0.001
Outline of the supra-orbital region. norma verticalis	1	4.16	0.001	Outline of the supra-orbital region. norma verticalis	1	5.42	0.001
Sharply angulated occipital. norma lateralis	2	3.65	0.001	Tuber parietale	2	5.37	0.001
Torus occipitalis transversus	2	3.59	0.001	Articular tubercle configuration	3	4.98	0.001
Sagittal keel on the frontal	2	3.09	0.001	Sagittal keel on the frontal	3	4.64	0.001
Torus angularis parietalis	2	3.09	0.001	Torus angularis parietalis	2	4.64	0.001
Articular tubercle configuration	1	2.87	0.002	Sulcus postorbitalis	2	4.60	0.001
Crista supramastoidea continues with the processus zygomaticus temporalis	2	2.87	0.002	Crista supramastoidea continues with the processus zygomaticus temporalis	1	4.60	0.001
Petro-tympanic crest orientation	3	2.82	0.002	Sulcus supratoralis	3	4.60	0.001
Occipital bun	2	2.76	0.003	Postorbital constriction	3	4.60	0.001
Torus occipitalis transversus form in norma occipitalis	2	2.66	0.003	Projection of the supra-orbital region	2	4.51	0.001
Tuberculum supramastoideum anterius	2	2.66	0.003	Tuber frontale	2	4.34	0.001
Linea temporalis forming a crest on the frontal	3	2.64	0.003	Sharply angulated occipital. norma lateralis	2	4.00	0.001
Tuberculum zygomaticum posterius	2	2.62	0.004	Supramastoid groove	2	3.84	0.001
Tuber parietale	2	2.53	0.006	Temporal squama height	1	3.42	0.001
Sulcus supratoralis	2	2.39	0.009	Supra-orbital region: sulcus supraorbitalis	2	3.39	0.001
-	-	-	-	Torus occipitalis transversus form	2	3.39	0.001
-	-	-	-	Tuberculum supramastoideum anterius	3	2.71	0.003
-	-	-	-	Supra-orbital region: sulcus supraorbitalis	2	2.64	0.004
-	-	-	-	Development of the crista supramastoidea at the porion	3	2.48	0.0007
-	-	-	-	Torus occipitalis transversus form	3	2.40	0.008
-	-	-	-	Outline of the supra-orbital region. norma facialis	1	2.40	00008

The statistical analysis identifies the character states that contribute the most to the formation of each class. The T-Value (pertinence criterion) must be ≥2 at *p*<0.05.

## Discussion

Overall, both the geometric morphometric analysis and the scoring of morphological features included in our phenetic study support the uniqueness of *H. heidelbergensis* as a species that was distributed on a wide geographical horizon, including Eurasia and Africa; at the same time, it was a rather polymorphic taxon and was probably ancestral to the origin of both the Neandertals and our own species, *H. sapiens*.

The DFA based on geometric morphometrics and Procrustes analysis (Figure 1) distinguishes between the three pre-defined groups: Early Pleistocene humans (*H. habilis*, D2280 and D2700 from Dmanisi, *H. ergaster*, *H. erectus*), Neandertals (*H. neanderthalensis*), and modern humans (*H. sapiens*). Along the first axis (80.9% of variance), we observe an overall consistency with their respective chronologies and phylogenetic positions. The empty chronological space between the Early Pleistocene archaic sample and the more recent as well as derived groups (Neandertals and modern humans) is filled by Mid-Pleistocene specimens, including Ceprano.

However, the morphological space identified by the cranial shape of Ceprano does not show clear affinities with other Mid-Pleistocene fossils. Along the first discriminant function it appears close to the Early Pleistocene sample attributed to *H. erectus* and, particularly, to Asian fossils such as Sangiran 17 (near Ceprano along the second discriminant function as well). The other Asian specimens (Mid-Pleistocene fossils from Zhoukoudian, China, and Late Pleistocene specimens from Ngandong, Java) also exhibit affinities with fossils dated to the Early Pleistocene. Despite the observed differences in shape between the Asian sample and the African fossils attributed to *H. ergaster* (e.g. widening of the braincase at the *stephanion* level), it is difficult to conclude whether these differences are able to distinguish Asian and African specimens as separate taxa with the widespread and highly variable taxon referred to as *H. erectus*. Nevertheless, the main result from this geometric morphometrics analysis supports previous claims that Ceprano definitively displays an “*erectus*-like” morphological architecture [4], [14].

By contrast, other Mid-Pleistocene specimens show different morphological trends. particularly – disregarding the position of Steinheim and Jinniu Shan, which seems to reflect the important deformations affecting both these specimens [22], [23] – fossils like SH5, Petralona, Dali, and Kabwe share a similar position in the shape space, somewhat midway between *H. erectus* and *H. neanderthalensis*.

We should conclude that Ceprano is characterized by an archaic shape. At the same time, however, it shows a peculiar combination of discrete features [5], as the second part of our protocol demonstrates (Figure 2).

Similarly to the previous analysis, the discrete features succeeded in identifying different taxonomic entities (Figure 2). Asian and African specimens of the Early Pleistocene show regional differences, supporting the existence of two distinct taxa or, at least, two diverging evolutionary lineages: *H. erectus* sensu stricto in Asia and *H. ergaster* in Africa [24]. The *H. sapiens* group includes the Qafzeh/Skhūl sample and “pre-modern” late Mid-Pleistocene specimens from Africa (Omo II, LH18, Singa, and Jebel Irhoud 1). Although this study does not focus on Early Pleistocene *Homo* or on the origin of modern humans, we note that these results support the validity of our phenetic approach.

**Figure 3:**
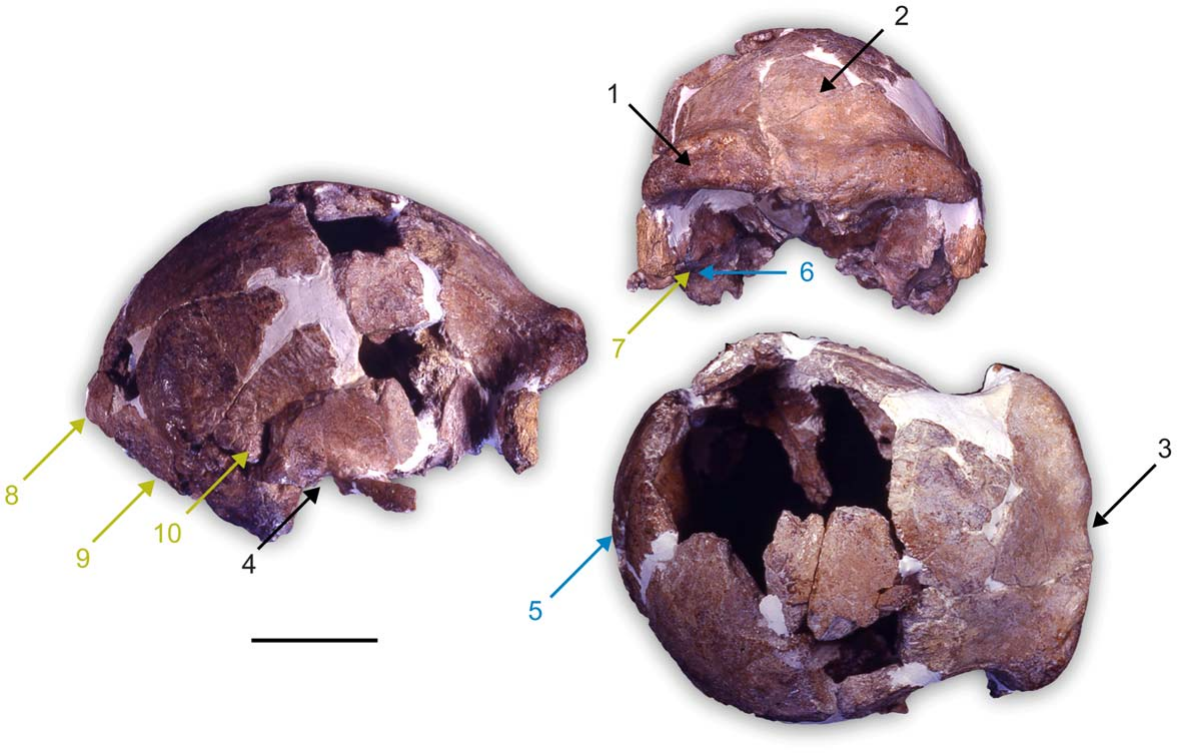
Statistically significant traits that describe the Mid-Pleistocene cluster including Ceprano. Numbers represent the following morphological features: Features 1 to 4 (black) traits that are more exclusive of Mid-Pleistocene specimens (i.e. 1: incomplete sulcus supraorbitalis, 2: frontal tuber weakly developed medially shifted, 3: supraorbital region medially concave, 4: intermediate position of the external auditory meatus in regard to the processus zygomaticus temporalis); 5 and 6 (blue)  =  more derived traits (i.e. 5: straight torus occipitalis transversus, 6: medio-lateral concavity of the articular tubercle); 7 to 10 (green)  =  more primitive traits (i.e. 7: petro-tympanic crest orientated downward, 8: opisthocranion coincident with inion, 9: processus retromastoideus, 10: torus angularis parietalis). Pertinence criterion for statistical significance: T-values>2, *p*<0.05. Scale bar  = 50 mm.

From this analysis it is clear that Ceprano displays a unique combination of morphological features (Figure 3). It shows traits that are found among Mid-Pleistocene specimens (e.g. frontal tuber weakly developed and medially positioned, supraorbital region medially concave, incomplete sulcus supraorbitalis, intermediate position of the external auditory meatus in regard to the processus zygomaticus temporalis, see, Table 3), but also traits that are considered as derived (straight torus occipitalis transversus, medio-lateral concavity of the articular tubercle) [25]. However, none of these are really Neandertal autapomorphic features, since they are widespread in Eurasia during the Middle Pleistocene. Actually, most of the Neandertal morphological features occur in the upper face of the European Mid-Pleistocene humans [25], [26], but in Ceprano only the suborbital region can be examined and it does not show any Neandertal resemblance. Conversely, Ceprano exhibits features that are common among *H. erectus* and/or *H. ergaster* (e.g. torus angularis parietalis, petro-tympanic crest orientated downward, processus retromastoideus and opisthocranion coincident with inion) [27], [28]. The result of this peculiar morphology is that Ceprano clusters in our analysis with other European, African and Asian Mid-Pleistocene specimens – such as Petralona, Dali, Kabwe, Jinniu Shan, Steinheim, and SH5 – furnishing a rather plesiomorphic phenetic link among them.

On the basis of this morphological affinity, it seems appropriate to group Ceprano with these fossils, and consider them as a single taxon. The available nomen for this putative species is *H. heidelbergensis* [15], whose distinctiveness stands on the retention of a number of archaic traits combined with features that are more derived and independent from any Neandertal ancestry. Especially, the morphology of the frontal bone seems to bear most of these traits (shape of supraorbital torus and occurrence of frontal tuber, in particular) [5], [13]. This conclusion is further supported by the position of the Ethiopian calvarium known as Daka in the analysis, where it emerges as part of the *H. ergaster* cluster along with OH9 and other African specimens (Figure 2). This result would suggest that *H. ergaster* survived as a distinct species until 1 Ma, and would discard the validity of the species *H. cepranensis* [11], which was based on the claimed affinities between Daka and Ceprano that we did not observe. At the same time, it should be noted that the mandible AT-888 associated with the SH5 cranium from Atapuerca has been shown to share affinities with the holotype of *H. heidelbergensis*: the Mauer mandible [18], [29]. Thus we can include the so-called “Ante-Neandertals” from Europe in the same taxonomical unit with other Mid-Pleistocene samples from Africa and continental Asia.

Combining the results of the two approaches of our phenetic analysis, Ceprano should be reasonably accommodated as part of a Mid-Pleistocene human taxon *H. heidelbergensis*, which would include European, African, and Asian specimens. Moreover, the combination of archaic and derived features exhibited by the Italian specimen represents a “node” connecting the different poles of such a polymorphic humanity. In this respect, it appears of particular interest that:

1) Ceprano shows strong morphological affinities with extra-European Mid-Pleistocene specimens, even more than with many of its European counterparts, supporting the above-mentioned conclusion of a widespread single species;

2) This morphological proximity suggests a dispersal that occurred approximately around the Early/Middle Pleistocene boundary (0.780 Ma), in Africa and Eurasia, and that is referable to a single species of derived (i.e. encephalized) humans;

3) Ceprano combines a rather primitive architecture of the braincase with derived features, thus possibly representing the ancestral (i.e. the most archaic-looking) morphotype of this taxon distributed both in Africa and Eurasia;

4) From this perspective, the morphology of Ceprano constitutes a phylogenetic “bridge” between Early Pleistocene *Homo* representatives and related forms (*H. erectus* sensu lato), and more recent and derived populations included in the species *H. heidelbergensis*;

5) These conclusions are also coherent with the new chronology proposed for Ceprano (ranging between 430 and 385 ka, [8]), when assuming a great variability in the Middle Pleistocene of Europe, with the occurrence of, some populations or single individuals that exhibit retention of a more archaic shape while others appear more derived [30].

In sum, our analysis demonstrates that Ceprano, as a calvarium, could represent an appropriate “counterpart” of, the holotype of *Homo heidelbergensis* (the mandible from Mauer [15]), bringing together both features observed on the human samples of the Middle Pleistocene referred to this widespread species and plesiomorphic traits shared with earlier or more archaic humans.

## Materials and Methods

### Material

The fossil sample was selected in order to encompass as much of the Pleistocene fossil record as possible. 39 fossils from Africa, Asia, and Europe were studied (Early Pleistocene: 9, Middle Pleistocene: 14 among which Ceprano, Neandertals: 9 late *H. erectus*: 3, *H. sapiens*: 7) (Table 1). Additionally, 68 modern humans from Africa, Europe and Asia (18 Neolithics, 50 extant modern humans) were included: 1- provide a sufficient sample of modern humans spread out over a span of time similar to that of the Neandertals (i.e. 130,000 years); 2- take into account the margin of error in dating the fossil sample; and 3- test the reliability of the character data set and the statistical method used in the study. Considering the continuing debates in palaeoanthropology over taxon recognition, the fossils were grouped according to their relative chronological position, with the exception of *H. neanderthalensis* and *H. sapiens* for which a relative consensus exists even though the debate is not totally closed [31], [32]. No a priori species grouping were used. Finally, no juveniles were included in the study with the exception of D2700 due to the scarce fossil sample available for Early Pleistocene.

### Methods

Geometric morphometrics shape analysis (see, [33]) is based on 14 landmarks (Figure S1, Table S3) chosen to describe at best the calvarium morphology, while taking into consideration the state of preservation of the fossils. We ran a Generalized Procrustes Analysis, a Principal Component Analysis (PCA) based on the procrustes residuals and a Discriminant Function Analysis (DFA) to discriminate three pre-defined groups (modern humans, Neandertals and Early Pleistocene specimens). This analysis uses the first 6 Principle Components (PC) (80.56% of the total variance, see, Tables S4 and S7) since the number of variables must be lower than 7 (smallest group number of specimens). The discrimination between these groups is used as a “pattern” to study fossil of interests which are introduced *a posteriori* in the analysis. The Wilks' lambda statistics [34] (see, Table S6), used to validate the discrimination, necessitates covariance matrices equality of each group which can be test using a Box's M test [35] (Table S5). In order to run this test we need to randomly select 14 modern humans to obtain groups, which are of comparable size (i.e. number of individuals, see Table S2). Additionally, we tested the impact of size on specimens shape modifications in order to identify a possible allometric trend in our data. We used linear regression, which was calculated for each PCs involved in the computation of the discriminant functions when compared to centroid size [36]. We used Morphologika 2 v2.5 [37] (APG, ACP, linear regression) and SPSS v11.5 ©SPSS Inc. 1989-2002 (linear Discriminant Function Analysis).

#### Hierarchical classification

50 morphological traits (Figure S2, Tables S9 and S12) were selected from the literature [25], [27], [28], [38]–[43] in order to encompass most of Pleistocene *Homo* sp. variation. Features that were too often missing and that did not meet the standard of repeatability were discarded (see test of intra-observer repeatability, Tables S10 and S11). We used two types of variables: binary (absence “1”/presence “2”) and continuous variables (divided qualitatively into 3 character states). Character states were not polarized phylogenetically. Specimens' description occurred through three sessions by each worker and observations were compared to published data. The phenetic analysis, as recently described [18], uses morphological distance matrices between specimens (multiple correspondence analysis, chi-square metric) to build dendrograms (hierarchical classification, ward's criterion [44]). Clusters from the dendrogram are consolidated to obtain the best classification (see, Tables S13 and S14). Each group from the dendrogram are described by statistically significant morphological features (pertinence criterion: T-Value>2, *p*<0.05). The variables do not have to be independent. No *a priori* groups were specified before the beginning of the analysis. Two analyses were run: the global analysis based on the 50 morphological features and the partial analysis which allows the inclusion of 8 additional specimens (Qafzeh 9, Gibraltar 1, La Quina H5, Ngawi 1, OH9, D2280, D2700 and Daka). Thirteen morphological features (2, 10, 14, 15, 16, 18, 25, 26, 40, 41, 43, 47, 50) not preserved on these specimens were not used in this analysis. We used SPAD (v5.5 ©DECISIA 1996–2002).

## Supporting Information

Figure S1 **Landmarks used in the geometric morphometrics analysis.** (Spy 1 © IRSNB, Bruxelles, Belgique). Description of each landmark can be found in Table S3.
(TIF)

Figure S2 **Morphological features included in the study** **(Abri Pataud).** Each number designates a morphological trait which description can be found in Table S9.
(TIF)

Figure S3 **Full dendrograms from hierarchical classification based on discrete features: general (A) and partial (B) analyses.** Branches and number at nodes express morphological distance between clusters. Clusters are built thanks to Ward's criterion. Modern humans are clearly separated from the fossils in both dendrograms to the exception of African late Mid-Pleistocene specimens Jebel Irhoud 1, Omo II and LH18. Neandertals form a cluster which includes the pre-Neandertal Gibraltar 1. Early Pleistocene specimens and Asian specimens often referred to *H. erectus* sensu stricto form two separated sub-groups in the same larger cluster. A) Ceprano is part of a Mid-Pleistocene cluster with African and Eurasian fossils. B) Partial analysis allows the inclusion of 8 additional specimens (Qafzeh 9, Gibraltar 1, La Quina H5, Ngawi 1, OH9, D2280, D2700 and Daka). 13 morphological features (2, 10, 14, 15, 16, 18, 25, 26, 40, 41, 43, 47, 50) not preserved on these specimens are not used in this analysis. Again, Ceprano is included in a Mid-Pleistocene cluster along with African and Eurasian fossils.
(TIF)

Table S1 **Details of the Historic and Neolithic specimens.** Period, denomination, number of male and female individuals and total number of specimens.
(DOC)

Table S2 **Holocene specimens included in the geometric morphometrics analysis.** Due to the conservation state of the Saharan series from Hassi El Abiod, no female individual were included.
(DOC)

Table S3 **Landmarks used in for the geometric morphometrics analysis.** Number. name. description and type for each landmark.
(DOC)

Table S4 **Main Principal Components from the procrustes shape analysis.** Eigenvalues. percentage of variance and percentage of cumulated variance for each principal component.
(DOC)

Table S5 **Discriminant Function Analysis: Box's M results on the covariance matrices of the three predefined groups.** Covariance matrices of the three groups are considered equals.
(DOC)

Table S6 **Discriminant Function Analysis: quality of the discrimination.** The Wilks' lambda results validate the discrimination of each function at *p*<0.0001.
(DOC)

Table S7 **Discriminant Function Analysis: Principal Component contribution to each discriminant function and coefficient for each function.** CP1 contributes the most to the first discriminant function while CP2 contributes the most to the second discriminant function.
(DOC)

Table S8 **Linear regression results for the six first principal components when compared to centroïde size.** The six first PCs are involved in the computation of the discriminant functions. None of the R^2^ and F values are significant. Thus, the centroid size does not seem to have a significant impact on the specimens' shape.
(DOC)

Table S9 **Morphological features and character states used in the phenetic analyses.** Morphological traits and character states used in the study; the 50 features were selected after a morphological survey of qualitative features of the calvaria in literature.
(DOC)

Table S10 **Frequency distribution of the morphological traits of the study for three repetitions by the same observer.** N  =  number of unobservable traits; * marks morphological features with intra-observer errors.
(DOC)

Table S11 **Chi-square test values for the comparisons between repetitions by the same observer.** * These 15 morphological features were found, during the intra-observer test, to show differences among trials. A chi-square test (with a Yates correction for small sample size when appropriate) was used to determine if the differences were significant. None of the tests in this table are statistically significant.
(DOC)

Table S12 **Character states for each morphological features and each specimens of the study.** First line numbers indicate morphological features, other lines numbers indicate the character state for each trait for each specimen, (-) indicates missing data (see Table S7).
(DOC)

Table S13 **Hierarchical classification: general analysis (Figure S3A); classification consolidation through iterations.** In successive iterations, the probability of the partition (i.e., the clustering process) is adjusted (i.e., individuals are randomly reallocated to the different clusters) to maximize the likelihood of the data given the number of clusters. The number of iterations is lower than 10 and the inertia does not vary. The partition is thus, solid.
(DOC)

Table S14 **Hierarchical classification: partial analysis (Figure S3B); classification consolidation through iterations.** In successive iterations, the probability of the partition (i.e., the clustering process) is adjusted (i.e., individuals are randomly reallocated to the different clusters) to maximize the likelihood of the data given the number of clusters. The number of iterations is lower than 10 and the inertia does not vary. The partition is thus, solid.
(DOC)

Table S15 **Hierarchical classification, general analysis (Figure S3A): description of modern Humans and Neandertals clusters by the most relevant morphological features and character states.** The statistical analysis identifies the character states that contribute the most to the formation of each class. The T-Value (pertinence criterion) must be ≥2 at *p*<0.05.
(DOC)
