# Supplementary figures and images for: The Stem Species of Our Species: A Place for the Archaic Human Cranium from Ceprano, Italy
Source: PLoS One. 2011 Apr 20;6(4):e18821. doi: 10.1371/journal.pone.0018821 (PMC3080388; doi:10.1371/journal.pone.0018821)

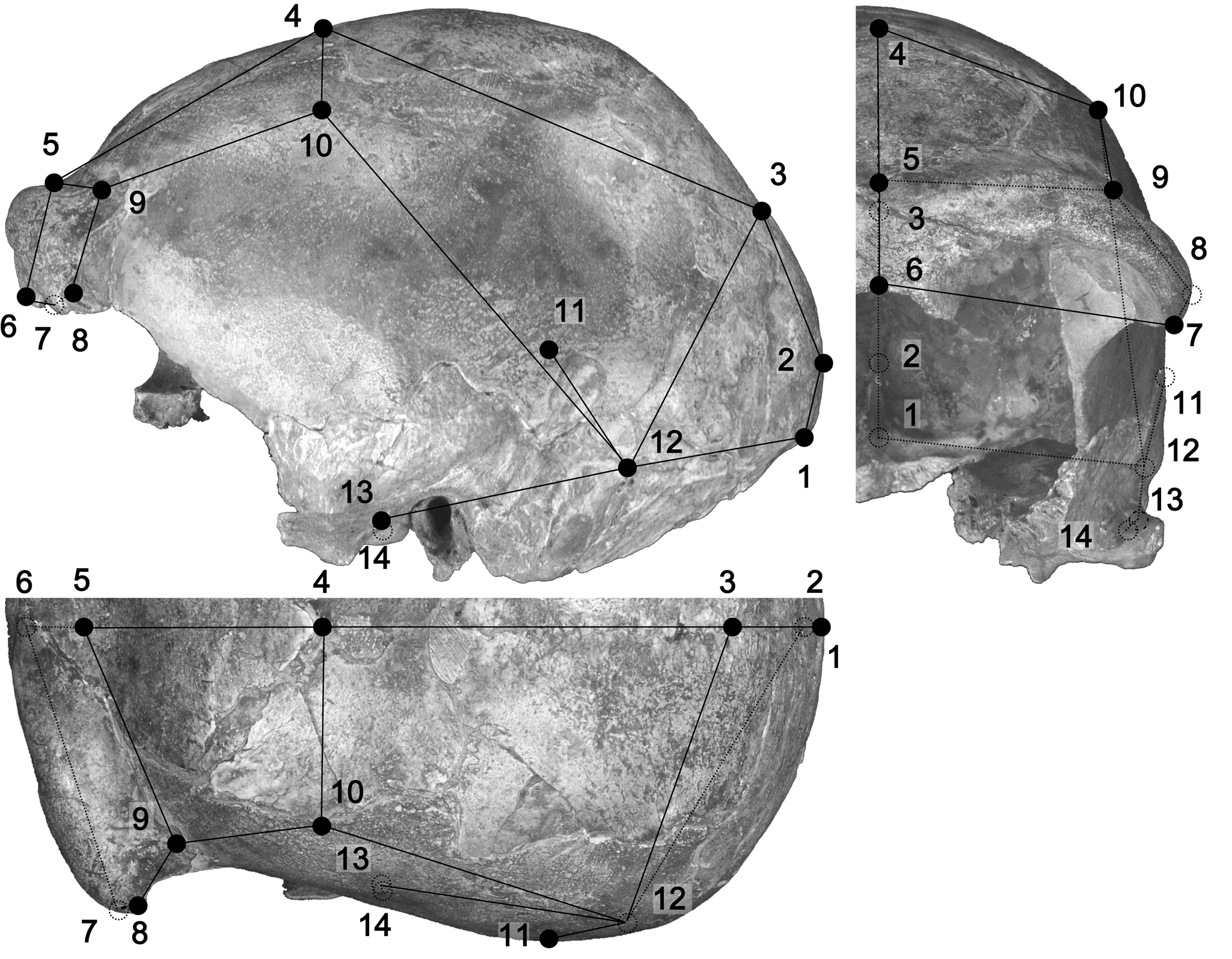

Supplement: Figure S1 — Landmarks used in the geometric morphometrics analysis. (Spy 1 © IRSNB, Bruxelles, Belgique). Description of each landmark can be found in Table S3. (TIF) [file pone.0018821.s001.tif]

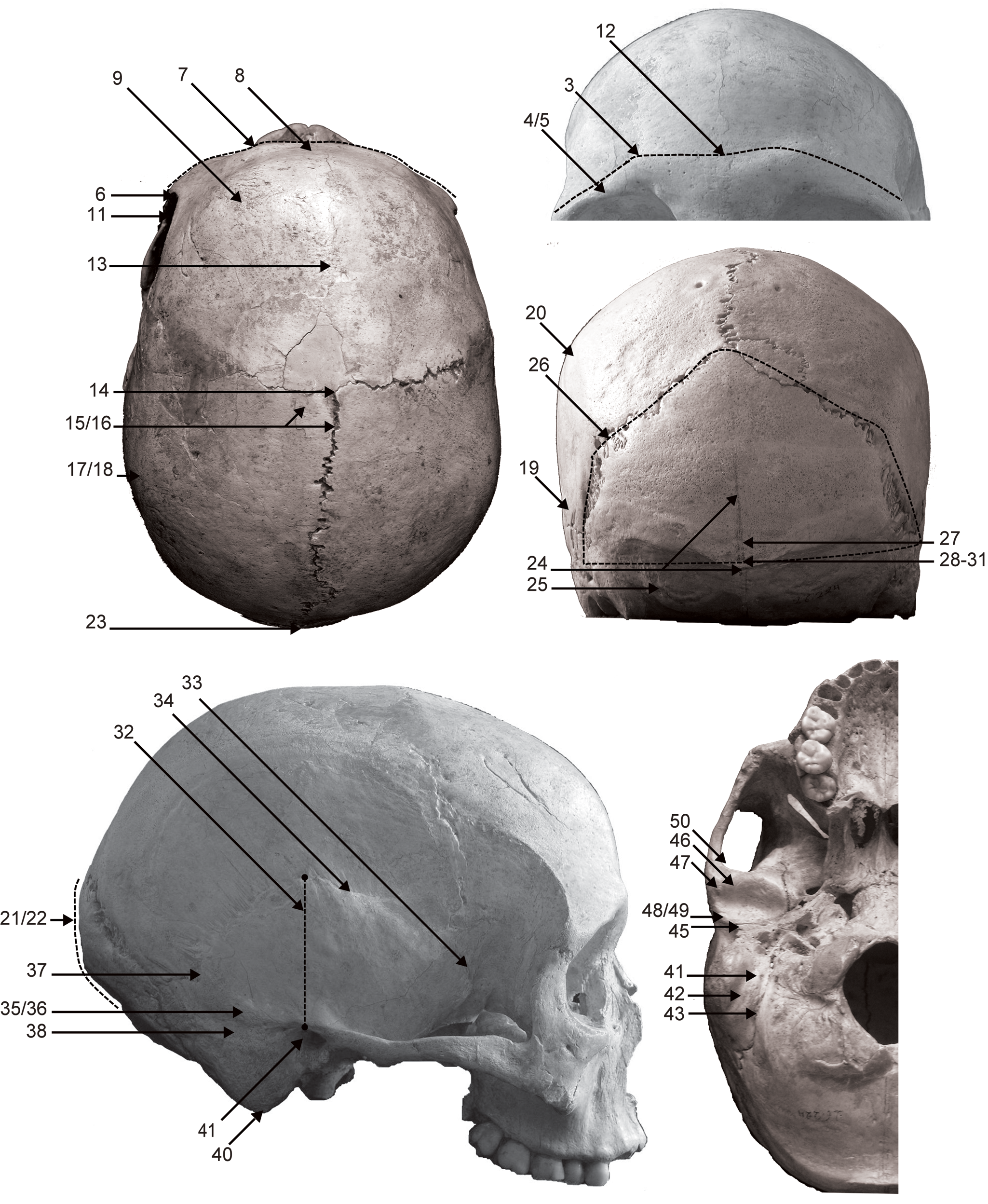

Supplement: Figure S2 — Morphological features included in the study (Abri Pataud). Each number designates a morphological trait which description can be found in Table S9. (TIF) [file pone.0018821.s002.tif]

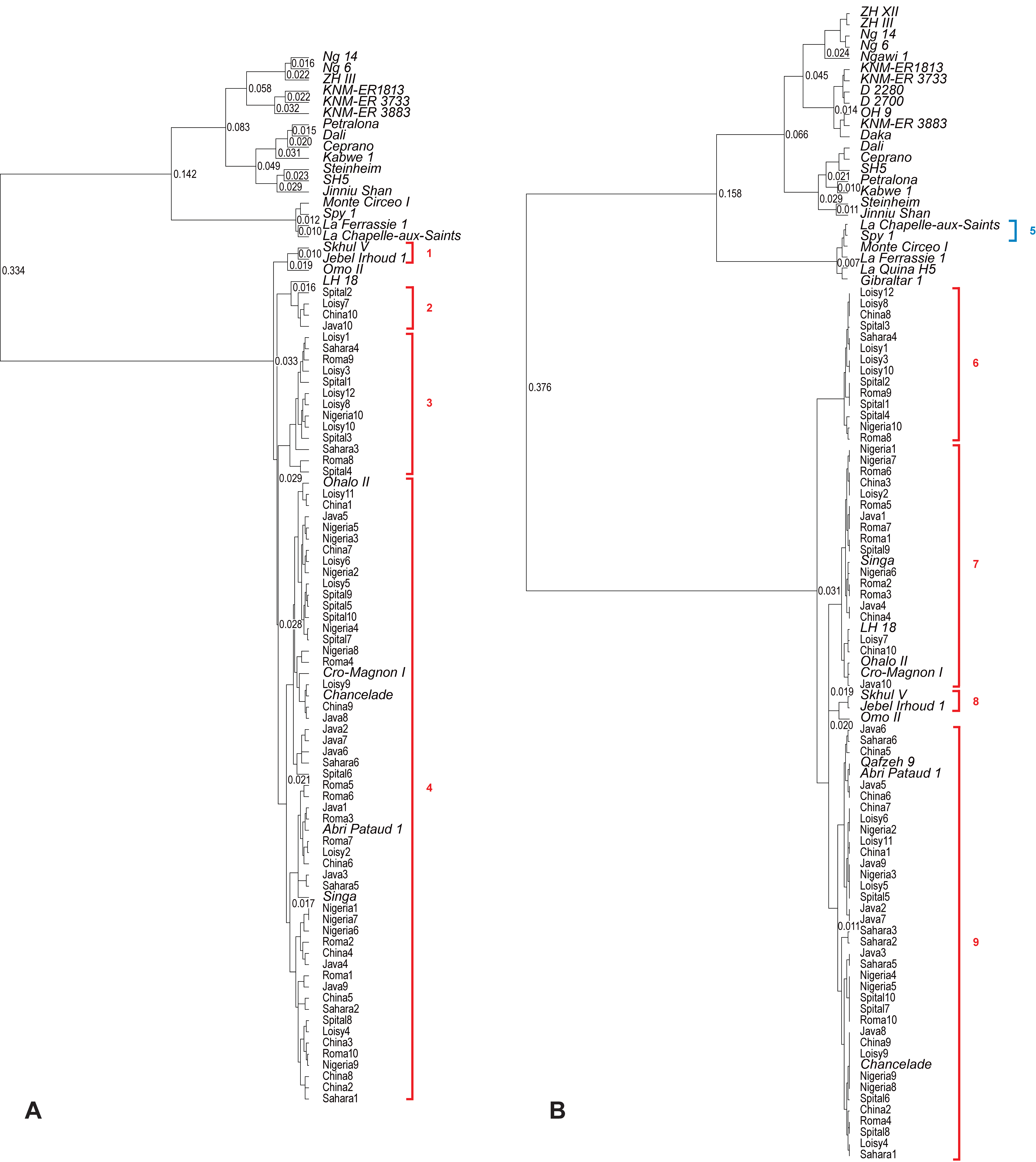

Supplement: Figure S3 — Full dendrograms from hierarchical classification based on discrete features: general (A) and partial (B) analyses. Branches and number at nodes express morphological distance between clusters. Clusters are built thanks to Ward's criterion. Modern humans are clearly separated from the fossils in both dendrograms to the exception of African late Mid-Pleistocene specimens Jebel Irhoud 1, Omo II and LH18. Neandertals form a cluster which includes the pre-Neandertal Gibraltar 1. Early Pleistocene specimens and Asian specimens often referred to H. erectus sensu stricto form two separated sub-groups in the same larger cluster. A) Ceprano is part of a Mid-Pleistocene cluster with African and Eurasian fossils. B) Partial analysis allows the inclusion of 8 additional specimens (Qafzeh 9, Gibraltar 1, La Quina H5, Ngawi 1, OH9, D2280, D2700 and Daka). 13 morphological features (2, 10, 14, 15, 16, 18, 25, 26, 40, 41, 43, 47, 50) not preserved on these specimens are not used in this analysis. Again, Ceprano is included in a Mid-Pleistocene cluster along with African and Eurasian fossils. (TIF) [file pone.0018821.s003.tif]
