# Supplementary material for: The Stem Species of Our Species: A Place for the Archaic Human Cranium from Ceprano, Italy
Source: PLoS One. 2011 Apr 20;6(4):e18821. doi: 10.1371/journal.pone.0018821 (PMC3080388; doi:10.1371/journal.pone.0018821)
Supplement: Table S1 — Details of the Historic and Neolithic specimens. Period, denomination, number of male and female individuals and total number of specimens. (DOC) [file pone.0018821.s004.doc]

**Table S1.**

| **Series** | **Period** | **Label** | **Male** | **Female** | **Unknown** | **Total** |
| --- | --- | --- | --- | --- | --- | --- |
| **Hassi-el-Abiod** | 6970 bp ± 130 | Sahara | 5 | - | 1 | 6 |
| **Loisy-en-Brie** | 3740 bp ± 120 | Loisy | - | - | 12 | 12 |
| **Spitalfields** | 17^th^-19^th^ centuries | Spital | 5 | 5 | - | 10 |
| **Romania** | 19^th^ century | Rouma | 5 | 5 | - | 10 |
| **China** | 20^th^ century | China | 8 | 2 | - | 10 |
| **Java** | 20^th^ century | Java | 5 | 5 | - | 10 |
| **Nigeria** | 20^th^ century | Nigeria | 3 | 2 | 5 | 10 |
| **Total** | - | - | 31 | 19 | 18 | 68 |
