# Supplementary material for: The Stem Species of Our Species: A Place for the Archaic Human Cranium from Ceprano, Italy
Source: PLoS One. 2011 Apr 20;6(4):e18821. doi: 10.1371/journal.pone.0018821 (PMC3080388; doi:10.1371/journal.pone.0018821)
Supplement: Table S2 — Holocene specimens included in the geometric morphometrics analysis. Due to the conservation state of the Saharan series from Hassi El Abiod, no female individual were included. (DOC) [file pone.0018821.s005.doc]

**Table S2.**

| **Europe** | | | **Africa** | | | **Asia** | | |
| --- | --- | --- | --- | --- | --- | --- | --- | --- |
| **specimen** | **chronology** | **sex** | **specimen** | **chronology** | **sex** | **specimen** | **chronology** | **sex** |
| Rouma12 | 19^th^ century | H | Nigeria2 | 19^th^ century | F | China4 | 19^th^ century | H |
| Rouma15 | 19^th^ century | F | Nigeria12 | 19^th^ century | H | China5 | 19^th^ century | F |
| Spital12 | 17^th^-19^th^ centuries | H | Sahara4 | 6970 bp ± 130 | H | Java9 | 19^th^ century | F |
| Spital28 | 17^th^-19^th^ centuries | F | Sahara9 | 6970 bp ± 130 | H | Java14 | 19^th^ century | H |
| Loisy10 | 3740 bp ± 120 | - | Sahara13 | 6970 bp ± 130 | H | - | - | - |
| Loisy12 | 3740 bp ± 120 | - |  |  |  |  |  |  |
