# Supplementary material for: The Stem Species of Our Species: A Place for the Archaic Human Cranium from Ceprano, Italy
Source: PLoS One. 2011 Apr 20;6(4):e18821. doi: 10.1371/journal.pone.0018821 (PMC3080388; doi:10.1371/journal.pone.0018821)
Supplement: Table S3 — Landmarks used in for the geometric morphometrics analysis. Number. name. description and type for each landmark. (DOC) [file pone.0018821.s006.doc]

**Table S3.**

| **n°** | **Name** | **description** | **type** |
| --- | --- | --- | --- |
| **1** | **Inion** | meeting point of the superior nuchal lines on the sagittal plan | II |
| **2** | **opisthocranion** | maximal length point of the cranium on the occipital | III |
| **3** | **Lambda** | parietal and occipital sutures meeting point | I |
| **4** | **Bregma** | posterior border of the frontal bone in the sagittal plane | I |
| **5** | **supra-glabellar** | point of maximum concavity behind supra-orbital torus in the midline | III |
| **6** | **Nasion** | naso-frontal suture and sagittal plan meeting point | I |
| **7** | **fronto-malare orbital** | lateral rim of the orbit and fronto-zygomatic suture meeting point | II |
| **8** | **fronto-malare temporal** | most postero-lateral point of the fronto-zygomatic suture | I |
| **9** | **fronto-temporale** | point where the temporal line reaches its most anteromedial position on the frontal | II |
| **10** | **Stephanion** | coronal suture and temporal line meeting point | I |
| **11** | **Euryon** | most lateral point of the lateral wall of the cranium | III |
| **12** | **Asterion** | parieto-temporal and lamboidal sutures meeting point | I |
| **13** | **Auriculae** | point at the maximum inflexion of the temporal zygomatic process | III |
| **14** | **Porion** | uppermost point on the margin of the external auditory meatus | III |
