# Supplementary material for: The Stem Species of Our Species: A Place for the Archaic Human Cranium from Ceprano, Italy
Source: PLoS One. 2011 Apr 20;6(4):e18821. doi: 10.1371/journal.pone.0018821 (PMC3080388; doi:10.1371/journal.pone.0018821)
Supplement: Table S4 — Main Principal Components from the procrustes shape analysis. Eigenvalues. percentage of variance and percentage of cumulated variance for each principal component. (DOC) [file pone.0018821.s007.doc]

**Table S4.**

| **PC** | **Eigen values** | **% total variance** | **% cumulated variance** |
| --- | --- | --- | --- |
| 1 | 0.00745 | 50.13 | 50.13 |
| 2 | 0.00122 | 8.21 | 58.34 |
| 3 | 0.00104 | 7.01 | 65.35 |
| 4 | 0.00098 | 6.59 | 71.94 |
| 5 | 0.00074 | 4.98 | 76.92 |
| 6 | 0.00054 | 3.64 | 80.56 |
| 7 | 0.00049 | 3.29 | 83.85 |
| 8 | 0.00033 | 2.23 | 86.08 |
| 9 | 0.00028 | 1.88 | 87.96 |
| 10 | 0.00023 | 1.56 | 89.52 |
| 11 | 0.00021 | 1.42 | 90.94 |
