# Supplementary material for: The Stem Species of Our Species: A Place for the Archaic Human Cranium from Ceprano, Italy
Source: PLoS One. 2011 Apr 20;6(4):e18821. doi: 10.1371/journal.pone.0018821 (PMC3080388; doi:10.1371/journal.pone.0018821)
Supplement: Table S5 — Discriminant Function Analysis: Box's M results on the covariance matrices of the three predefined groups. Covariance matrices of the three groups are considered equals. (DOC) [file pone.0018821.s008.doc]

**Table S5.**

| **Box’s M** |  | **68.660** |
| --- | --- | --- |
| F |  | 0.945 |
|  | df 1 | 42 |
|  | df 2 | 860.990 |
|  | p | 0.572 |
