# Supplementary material for: The Stem Species of Our Species: A Place for the Archaic Human Cranium from Ceprano, Italy
Source: PLoS One. 2011 Apr 20;6(4):e18821. doi: 10.1371/journal.pone.0018821 (PMC3080388; doi:10.1371/journal.pone.0018821)
Supplement: Table S6 — Discriminant Function Analysis: quality of the discrimination. The Wilks' lambda results validate the discrimination of each function at p<0.0001. (DOC) [file pone.0018821.s009.doc]

**Table S6**.

| **Functions** | **Wilks’ lambda** | **Chi square** | **df** | **p** | **eigenvalues** | **% of variance** | **Canonical correlation** |
| --- | --- | --- | --- | --- | --- | --- | --- |
| 1 | 0.030 | 103.894 | 12 | 0.0001 | 9.452 | 80.9 | 0.951 |
| 2 | 0.309 | 34.663 | 5 | 0.0001 | 2.238 | 19.1 | 0.831 |
