# Supplementary material for: The Stem Species of Our Species: A Place for the Archaic Human Cranium from Ceprano, Italy
Source: PLoS One. 2011 Apr 20;6(4):e18821. doi: 10.1371/journal.pone.0018821 (PMC3080388; doi:10.1371/journal.pone.0018821)
Supplement: Table S7 — Discriminant Function Analysis: Principal Component contribution to each discriminant function and coefficient for each function. CP1 contributes the most to the first discriminant function while CP2 contributes the most to the second discriminant function. (DOC) [file pone.0018821.s010.doc]

**Table S7.**

| **PC** | **Contribution to the discriminant functions** | | **Discriminant functions coefficients** | |
| --- | --- | --- | --- | --- |
|  | **Functions** | | **Functions** | |
|  | **1** | **2** | **1** | **2** |
| **1** | 0.926 | -0.129 | 1.045 | -0.099 |
| **4** | 0.076 | 0.703 | 0.172 | 1.035 |
| **2** | -0.072 | 0.251 | -0.097 | 0.467 |
| **6** | 0.001 | 0.180 | 0.155 | 0.251 |
| **3** | 0.097 | 0.164 | 0.218 | 0.357 |
| **5** | -0.039 | -0.122 | 0.213 | -0.316 |
