# Supplementary material for: The Stem Species of Our Species: A Place for the Archaic Human Cranium from Ceprano, Italy
Source: PLoS One. 2011 Apr 20;6(4):e18821. doi: 10.1371/journal.pone.0018821 (PMC3080388; doi:10.1371/journal.pone.0018821)
Supplement: Table S8 — Linear regression results for the six first principal components when compared to centroïde size. The six first PCs are involved in the computation of the discriminant functions. None of the R2 and F values are significant. Thus, the centroid size does not seem to have a significant impact on the specimens' shape. (DOC) [file pone.0018821.s011.doc]

**Table S8.**

|  | **R²** | **F** | ***p*** |
| --- | --- | --- | --- |
| **PC1** | 0.013 | 0.663 | 0.419 |
| **PC2** | 0.041 | 2.122 | 0.151 |
| **PC3** | 0.001 | 0.019 | 0.891 |
| **PC4** | 0.055 | 2.925 | 0.094 |
| **PC5** | 0.057 | 3.016 | 0.089 |
| **PC6** | 0.008 | 0.409 | 0.525 |
