# Supplementary material for: The Stem Species of Our Species: A Place for the Archaic Human Cranium from Ceprano, Italy
Source: PLoS One. 2011 Apr 20;6(4):e18821. doi: 10.1371/journal.pone.0018821 (PMC3080388; doi:10.1371/journal.pone.0018821)
Supplement: Table S9 — Morphological features and character states used in the phenetic analyses. Morphological traits and character states used in the study; the 50 features were selected after a morphological survey of qualitative features of the calvaria in literature. (DOC) [file pone.0018821.s012.doc]

**Table S9.**

| **Morphological features** |  |  | **character states** |  | **Morphological features** |  |  | **character states** |  | **Morphological features** |  |  | **character states** |
| --- | --- | --- | --- | --- | --- | --- | --- | --- | --- | --- | --- | --- | --- |
| Outline of the calvaria, *norma lateralis* | **1** | **1** | triangular |  | *Linea temporalis* width of the temporal band | **17** | **1** | absent |  | Outline of the superior border of the squama | **34** | **1** | curved or sinuous |
|  |  | **2** | circular |  |  |  | **2** | narrow (<20mm) |  |  |  | **2** | straight |
|  |  | **3** | pentagonal |  |  |  | **3** | wide (>20mm) |  | Development of the *crista supramastoidea* at the porion | **35** | **1** | absent |
| Frontal cord length / parietal cord length | **2** | **1** | Frontal < Parietal |  | *Linea temporalis*: superior line position on parietal (porion-bregma arc) | **18** | **1** | high (Rlt>0.55) |  |  |  | **2** | weakly-marked |
|  |  | **2** | Frontal ≈ Parietal |  |  |  | **2** | medial (0.54>Rlt>0.46) |  |  |  | **3** | marked |
|  |  | **3** | Frontal > Parietal |  |  |  | **3** | low (Rlt<0.45) |  | *Crista supramastoidea /* *processus zygomaticus temporalis* | **36** | **1** | not lined up |
| Outline of the supra-orbital region, *norma facialis* | **3** | **1** | straight |  | *Torus angularis parietalis* | **19** | **1** | absent |  |  |  | **2** | lined up |
|  |  | **2** | convex |  |  |  | **2** | present |  | *Tuberculum supramastoideum anterius* | **37** | **1** | absent |
| Supra-orbital region: *sulcus supraorbitalis* | **4** | **1** | complete |  | *Tuber parietale* | **20** | **1** | absent |  |  |  | **2** | present |
|  |  | **2** | incomplete |  |  |  | **2** | present, medially shifted |  | Supramastoid groove (between *crista supramstoidea* and *crista mastoidea*) | **38** | **1** | absent |
|  |  | **3** | absent: *arcus superciliairis* and *supraorbitalis* merged |  |  |  | **3** | present, high position |  |  |  | **2** | present, closed anteriorly |
| Projection of the supra-orbital region | **5** | **1** | not projecting |  | Outline of the occipital, *norma lateralis* | **21** | **1** | rounded profile |  |  |  | **3** | present |
|  |  | **2** | *arcus superciliairis* only |  |  |  | **2** | sharply angulated |  | Position of the auditory meatus / *processus zygomaticus temporalis* | **39** | **1** | under |
|  |  | **3** | whole supra-orbital region |  | Outline of the *planum occipitale*, *norma lateralis* | **22** | **1** | no convexity |  |  |  | **2** | intermediate |
| Postorbital constriction (Ipc=M9/M43) | **6** | **1** | important (Ipc<0.75) |  |  |  | **2** | convexity |  |  |  | **3** | aligned |
|  |  | **2** | weak (0.75≥Ipc≥0.85) |  | Occipital bun | **23** | **1** | absent |  | *Processus mastoidus*: downward development / basicranium | **40** | **1** | no |
|  |  | **3** | absent (Ipc>0.85) |  |  |  | **2** | present |  |  |  | **2** | yes |
| Outline of the supra-orbital region, *norma verticalis* | **7** | **1** | medially concave (glabella) |  | Opisthocranion coincident with inion | **24** | **1** | yes |  | Juxtamastoid ridge development / *processus mastoidus* | **41** | **1** | less developed |
|  |  | **2** | straight |  |  |  | **2** | no |  |  |  | **2** | as developed |
|  |  | **3** | convex |  | *Processus retromastoideus* | **25** | **1** | absent |  |  |  | **3** | more developed |
| *Sulcus postorbitalis* | **8** | **1** | absent |  |  |  | **2** | present |  | Digastric groove: presence of a bony bridge | **42** | **1** | no |
|  |  | **2** | medially present |  | Outline of the *planum occipitalis*, *norma occipitalis* | **26** | **1** | triangular |  |  |  | **2** | yes |
|  |  | **3** | present continue |  |  |  | **2** | circular |  | *Crista occipitomastoidea* | **43** | **1** | absent |
| *Tuber frontale* | **9** | **1** | absent |  |  |  | **3** | pentagonal |  |  |  | **2** | present |
|  |  | **2** | defined, medially shifted |  | Suprainiac fossa | **27** | **1** | absent |  | Glenoid cavity depth / the articular tubercle lowest point, *norma lateralis* | **44** | **1** | shallow (<0.9mm) |
|  |  | **3** | present |  |  |  | **2** | weakly-delineated hollowing |  |  |  | **2** | deep (>0.9mm) |
| Antero-posterior convexity of the frontal (Ifc=M29*100/M26) | **10** | **1** | weak (Ifc≥0.95) |  |  |  | **3** | present |  | Petro-tympanic crest orientation in relation to the sagittal plan | **45** | **1** | perpendicular |
|  |  | **2** | average (95>Ifc≥90) |  | *Sulcus supratoralis* | **28** | **1** | absent |  |  |  | **2** | frontward |
|  |  | **3** | important (Ifc<90) |  |  |  | **2** | hollowing |  |  |  | **3** | downward |
| *Linea temporalis* forming a crest on the frontal | **11** | **1** | absent |  |  |  | **3** | present |  | Articular tubercle configuration | **46** | **1** | mediolateral concavity |
|  |  | **2** | present, unique crest |  | *Torus occipitalis transversus* | **29** | **1** | absent |  |  |  | **2** | anteroposterior convexity |
|  |  | **3** | present, double crests |  |  |  | **2** | present: medially protruding |  |  |  | **3** | mediolateral convexity and vertical |
| Medio-sagittal supra-glabellar tubercle (1) | **12** | **1** | absent |  |  |  | **3** | present: bilaterally protruding |  | *Tuberculum zygomaticum anterius* | **47** | **1** | absent to weakly-marked |
|  |  | **2** | present |  | *Torus occipitalis transversus* form, *norma occipitalis* | **30** | **1** | absent |  |  |  | **2** | marked |
| Sagittal keel on the frontal | **13** | **1** | absent |  |  |  | **2** | straight |  | *Tuberculum zygomaticum posterius* (post glenoid process) | **48** | **1** | absent or weakly-marked |
|  |  | **2** | present |  |  |  | **3** | convex |  |  |  | **2** | marked |
| Bregmatic eminence | **14** | **1** | absent |  | *Protuberantia occipitalis externa* | **31** | **1** | absent |  | Tympanal contribution to the posterior wall of the glenoid cavity | **49** | **1** | weak |
|  |  | **2** | present |  |  |  | **2** | present |  |  |  | **2** | important |
| Sagittal keel on the bregma-lambda arc | **15** | **1** | absent |  | Temporal squama height | **32** | **1** | low (Iet ≤ 60) |  | Preglenoid tubercle | **50** | **1** | absent |
|  |  | **2** | present |  |  |  | **2** | high (Iet > 60) |  |  |  | **2** | present |
| Parasagittal hollowing on both sides of the parietal suture | **16** | **1** | absent |  | Outline of the anterior border of the squama | **33** | **1** | curved or sinuous |  |  |  |  |  |
|  |  | **2** | present |  |  |  | **2** | straight |  |  |  |  |  |
