# Supplementary material for: The Stem Species of Our Species: A Place for the Archaic Human Cranium from Ceprano, Italy
Source: PLoS One. 2011 Apr 20;6(4):e18821. doi: 10.1371/journal.pone.0018821 (PMC3080388; doi:10.1371/journal.pone.0018821)
Supplement: Table S10 — Frequency distribution of the morphological traits of the study for three repetitions by the same observer. N = number of unobservable traits; * marks morphological features with intra-observer errors. (DOC) [file pone.0018821.s013.doc]

**Table S10.**

| **Repetitions** | | **I** | | | |  | **II** | | | |  | **III** | | |  |  |
| --- | --- | --- | --- | --- | --- | --- | --- | --- | --- | --- | --- | --- | --- | --- | --- | --- |
| **Traits states** | | **1** | **2** | **3** | **N** |  | **1** | **2** | **3** | **N** |  | **1** | **2** | **3** | **N** |  |
| **Traits** |  |  |  |  |  |  |  |  |  |  |  |  |  |  |  |  |
| **1** |  | 5 | 4 | 16 | 0 |  | 5 | 4 | 16 | 0 |  | 5 | 4 | 16 | 0 |  |
| **2** |  | 10 | 5 | 5 | 0 |  | 10 | 5 | 5 | 0 |  | 10 | 5 | 5 | 0 |  |
| **3** |  | 1 | 24 | - | 0 |  | 1 | 24 | - | 0 |  | 1 | 24 | - | 0 |  |
| **4** |  | 16 | 5 | 4 | 0 |  | 16 | 5 | 4 | 0 |  | 16 | 5 | 4 | 0 |  |
| **5** |  | 10 | 6 | 9 | 0 |  | 11 | 5 | 9 | 0 |  | 10 | 6 | 9 | 0 | ***** |
| **6** |  | 2 | 3 | 19 | 0 |  | 2 | 3 | 19 | 0 |  | 2 | 3 | 19 | 0 |  |
| **7** |  | 3 | 2 | 20 | 0 |  | 3 | 2 | 20 | 0 |  | 3 | 2 | 20 | 0 |  |
| **8** |  | 14 | 2 | 9 | 0 |  | 14 | 2 | 9 | 0 |  | 13 | 3 | 9 | 0 | ***** |
| **9** |  | 0 | 5 | 20 | 0 |  | 0 | 5 | 20 | 0 |  | 0 | 5 | 20 | 0 |  |
| **10** |  | 2 | 8 | 14 | 0 |  | 2 | 8 | 14 | 0 |  | 2 | 8 | 14 | 0 |  |
| **11** |  | 9 | 12 | 4 | 0 |  | 7 | 14 | 4 | 0 |  | 7 | 14 | 4 | 0 | ***** |
| **12** |  | 24 | 1 | - | 0 |  | 24 | 1 | - | 0 |  | 24 | 1 | - | 0 |  |
| **13** |  | 22 | 3 | - | 0 |  | 22 | 3 | - | 0 |  | 22 | 3 | - | 0 |  |
| **14** |  | 23 | 2 | - | 0 |  | 23 | 2 | - | 0 |  | 23 | 2 | - | 0 |  |
| **15** |  | 18 | 6 | - | 1 |  | 18 | 6 | - | 1 |  | 18 | 6 | - | 1 |  |
| **16** |  | 14 | 11 | - | 0 |  | 14 | 11 | - | 0 |  | 14 | 11 | - | 0 |  |
| **17** |  | 5 | 14 | 6 | 0 |  | 4 | 14 | 7 | 0 |  | 3 | 16 | 6 | 0 | ***** |
| **18** |  | 1 | 1 | 22 | 1 |  | 1 | 1 | 22 | 1 |  | 1 | 1 | 22 | 1 |  |
| **19** |  | 21 | 4 | - | 0 |  | 21 | 4 | - | 0 |  | 21 | 4 | - | 0 |  |
| **20** |  | 7 | 3 | 15 | 0 |  | 7 | 3 | 15 | 0 |  | 7 | 3 | 15 | 0 |  |
| **21** |  | 16 | 9 | - | 0 |  | 16 | 9 | - | 0 |  | 16 | 9 | - | 0 |  |
| **22** |  | 7 | 18 | - | 0 |  | 7 | 18 | - | 0 |  | 7 | 18 | - | 0 |  |
| **23** |  | 20 | 5 | - | 0 |  | 20 | 5 | - | 0 |  | 20 | 5 | - | 0 |  |
| **24** |  | 5 | 20 | - | 0 |  | 5 | 20 | - | 0 |  | 5 | 20 | - | 0 |  |
| **25** |  | 20 | 5 | - | 0 |  | 20 | 5 | - | 0 |  | 20 | 5 | - | 0 |  |
| **26** |  | 5 | 4 | 16 | 0 |  | 5 | 4 | 16 | 0 |  | 6 | 3 | 16 | 0 | ***** |
| **27** |  | 22 | 1 | 2 | 0 |  | 22 | 1 | 2 | 0 |  | 22 | 1 | 2 | 0 |  |
| **28** |  | 21 | 3 | 1 | 0 |  | 21 | 3 | 1 | 0 |  | 21 | 3 | 1 | 0 |  |
| **29** |  | 17 | 5 | 3 | 0 |  | 17 | 5 | 3 | 0 |  | 17 | 5 | 3 | 0 |  |
| **30** |  | 17 | 7 | 1 | 0 |  | 17 | 7 | 1 | 0 |  | 17 | 7 | 1 | 0 |  |
| **31** |  | 15 | 10 | - | 0 |  | 17 | 8 | - | 0 |  | 16 | 9 | - | 0 | ***** |
| **32** |  | 5 | 20 | - | 0 |  | 5 | 20 | - | 0 |  | 5 | 20 | - | 0 |  |
| **33** |  | 18 | 7 | - | 0 |  | 16 | 9 | - | 0 |  | 15 | 10 | - | 0 | ***** |
| **34** |  | 23 | 2 | - | 0 |  | 23 | 2 | - | 0 |  | 23 | 2 | - | 0 |  |
| **35** |  | 11 | 6 | 8 | 0 |  | 12 | 5 | 8 | 0 |  | 12 | 6 | 7 | 0 | ***** |
| **36** |  | 19 | 6 | - | 0 |  | 19 | 6 | - | 0 |  | 19 | 6 | - | 0 |  |
| **37** |  | 18 | 7 | - | 0 |  | 19 | 6 | - | 0 |  | 19 | 6 | - | 0 | ***** |
| **38** |  | 1 | 3 | 21 | 0 |  | 1 | 3 | 21 | 0 |  | 1 | 3 | 21 | 0 |  |
| **39** |  | 20 | 3 | 2 | 0 |  | 20 | 3 | 2 | 0 |  | 20 | 3 | 2 | 0 |  |
| **40** |  | 6 | 19 | - | 0 |  | 6 | 19 | - | 0 |  | 6 | 19 | - | 0 |  |
| **41** |  | 21 | 2 | 1 | 1 |  | 21 | 2 | 1 | 1 |  | 21 | 2 | 1 | 1 |  |
| **42** |  | 18 | 7 | - | 0 |  | 18 | 7 | - | 0 |  | 18 | 7 | - | 0 |  |
| **43** |  | 21 | 2 | - | 2 |  | 22 | 1 | - | 2 |  | 22 | 1 | - | 2 | ***** |
| **44** |  | 8 | 17 | - | 0 |  | 7 | 18 | - | 0 |  | 7 | 18 | - | 0 |  |
| **45** |  | 2 | 17 | 6 | 0 |  | 2 | 17 | 6 | 0 |  | 2 | 17 | 6 | 0 |  |
| **46** |  | 9 | 15 | 1 | 0 |  | 9 | 15 | 1 | 0 |  | 8 | 16 | 1 | 0 | ***** |
| **47** |  | 10 | 15 | - | 0 |  | 9 | 16 | - | 0 |  | 9 | 16 | - | 0 | ***** |
| **48** |  | 10 | 15 | - | 0 |  | 10 | 15 | - | 0 |  | 12 | 13 | - | 0 | ***** |
| **49** |  | 8 | 17 | - | 0 |  | 8 | 17 | - | 0 |  | 8 | 17 | - | 0 |  |
| **50** |  | 9 | 16 | - | 0 |  | 8 | 17 | - | 0 |  | 7 | 18 | - | 0 | ***** |
