# Supplementary material for: The Stem Species of Our Species: A Place for the Archaic Human Cranium from Ceprano, Italy
Source: PLoS One. 2011 Apr 20;6(4):e18821. doi: 10.1371/journal.pone.0018821 (PMC3080388; doi:10.1371/journal.pone.0018821)
Supplement: Table S11 — Chi-square test values for the comparisons between repetitions by the same observer. * These 15 morphological features were found, during the intra-observer test, to show differences among trials. A chi-square test (with a Yates correction for small sample size when appropriate) was used to determine if the differences were significant. None of the tests in this table are statistically significant. (DOC) [file pone.0018821.s014.doc]

**Table S11.**

| **Morphological features** |  | **Chi-square** | **p** | **df** |
| --- | --- | --- | --- | --- |
| Projection of the supra-orbital region | **5** | 0.182 | 0.996 | 4 |
| *Sulcus postorbitalis* | **8** | 0.334 | 0.987 | 4 |
| *Linea temporalis* forming a crest on the frontal | **11** | 0.548 | 0.969 | 4 |
| *Linea temporalis* width of the temporal band | **17** | 0.787 | 0.940 | 4 |
| Outline of the *planum occipitalis*, *norma occipitalis* | **26** | 0.307 | 0.989 | 4 |
| *Protuberantia occipitalis externa* | **31** | 0.347 | 0.841 | 2 |
| Outline of the anterior border of the squama | **33** | 1.417 | 0.492 | 2 |
| Development of the *crista supramastoidea* at the porion | **35** | 0.262 | 0.992 | 2 |
| *Tuberculum supramastoideum anterius* | **37** | 0.141 | 0.932 | 2 |
| *Crista occipitomastoidea* | **43** | 0.531 | 0.767 | 2 |
| Glenoid cavity depth / the articular tubercle lowest point, *norma lateralis* | **44** | 0.129 | 0.938 | 4 |
| Articular tubercle configuration | **46** | 0.120 | 0.998 | 2 |
| *Tuberculum zygomaticum anterius* | **47** | 0.114 | 0.945 | 2 |
| *Tuberculum zygomaticum posterius* (post glenoid process) | **48** | 0.436 | 0.894 | 2 |
| Preglenoid tubercle | **50** | 0.368 | 0.832 | 2 |
