# Supplementary material for: The Stem Species of Our Species: A Place for the Archaic Human Cranium from Ceprano, Italy
Source: PLoS One. 2011 Apr 20;6(4):e18821. doi: 10.1371/journal.pone.0018821 (PMC3080388; doi:10.1371/journal.pone.0018821)
Supplement: Table S12 — Character states for each morphological features and each specimens of the study. First line numbers indicate morphological features, other lines numbers indicate the character state for each trait for each specimen, (-) indicates missing data (see Table S7). (DOC) [file pone.0018821.s015.doc]

**Table S12.**

| **Morphological features** | **1** | **2** | **3** | **4** | **5** | **6** | **7** | **8** | **9** | **10** | **11** | **12** | **13** | **14** | **15** | **16** | **17** | **18** | **19** | **20** | **21** | **22** | **23** | **24** | **25** | **26** | **27** | **28** | **29** | **30** | **31** | **32** | **33** | **34** | **35** | **36** | **37** | **38** | **39** | **40** | **41** | **42** | **43** | **44** | **45** | **46** | **47** | **48** | **49** | **50** |
| --- | --- | --- | --- | --- | --- | --- | --- | --- | --- | --- | --- | --- | --- | --- | --- | --- | --- | --- | --- | --- | --- | --- | --- | --- | --- | --- | --- | --- | --- | --- | --- | --- | --- | --- | --- | --- | --- | --- | --- | --- | --- | --- | --- | --- | --- | --- | --- | --- | --- | --- |
| D 2280 | 1 | 3 | 2 | 2 | 3 | 1 | 2 | 3 | 1 | 2 | 2 | 1 | 2 | 2 | 2 | 1 | 2 | 1 | 2 | 1 | 1 | 2 | 1 | 1 | 1 | 1 | 1 | 3 | 2 | 2 | 2 | 1 | 1 | 1 | 2 | 2 | 1 | 2 | 1 | - | - | 2 | - | 2 | 3 | 3 | 1 | 2 | 2 | 2 |
| D 2700 | 1 | 2 | 2 | 2 | 2 | 1 | 3 | 3 | 1 | 2 | 2 | 1 | 2 | 2 | 2 | 2 | 2 | 1 | 2 | 1 | 1 | 1 | 1 | 1 | 1 | 2 | 1 | 1 | 1 | 1 | 1 | 2 | 1 | 2 | 1 | 2 | 1 | 2 | 1 | - | - | 1 | 1 | 1 | 3 | 1 | 1 | 2 | 2 | 1 |
| KNM-ER1813 | 1 | 3 | 2 | 2 | 3 | 1 | 2 | 3 | 2 | 2 | 2 | 1 | 1 | 1 | 1 | 1 | 2 | 1 | 1 | 1 | 2 | 1 | 1 | 1 | 1 | 1 | 1 | 2 | 2 | 2 | 1 | 1 | 2 | 1 | 3 | 2 | 1 | 2 | 1 | 1 | 2 | 2 | 1 | 1 | 3 | 1 | 1 | 2 | 1 | 1 |
| KNM-ER 3733 | 1 | 3 | 2 | 3 | 3 | 1 | 2 | 3 | 2 | 2 | 2 | 1 | 2 | 2 | 1 | 1 | 3 | 3 | 2 | 1 | 2 | 1 | 1 | 1 | 2 | 1 | 1 | 3 | 2 | 2 | 1 | 2 | 2 | 2 | 2 | 2 | 2 | 2 | 1 | 1 | 2 | 1 | 1 | 1 | 3 | 1 | 2 | 1 | 2 | 1 |
| KNM-ER 3883 | 1 | 3 | 2 | 3 | 3 | 1 | 3 | 3 | 2 | 1 | 2 | 1 | 1 | 2 | 2 | 1 | 3 | 3 | 1 | 1 | 2 | 2 | 1 | 2 | 1 | 2 | 1 | 2 | 2 | 3 | 1 | 2 | 2 | 2 | 2 | 2 | 1 | 3 | 1 | 2 | 1 | 1 | 1 | 2 | 3 | 1 | 2 | 2 | 2 | 2 |
| OH 9 | 1 | - | 2 | 3 | 3 | 1 | 1 | 3 | 2 | - | 2 | 1 | 1 | - | - | - | 2 | 2 | 1 | 1 | 2 | 2 | 1 | 1 | 1 | - | 1 | 2 | 2 | 2 | 2 | 1 | 1 | 2 | 2 | 2 | 1 | 2 | 1 | 2 | 1 | 2 | 2 | 2 | 3 | 3 | 2 | 1 | 2 | 1 |
| BOU-VP-2/66 | 1 | 3 | 2 | 2 | 3 | 1 | 1 | 3 | 2 | - | 2 | 1 | 2 | 1 | 2 | 2 | 3 | - | 2 | 2 | 2 | 2 | 1 | 1 | - | 1 | 1 | 1 | 1 | 1 | 2 | 2 | 2 | 1 | 2 | 2 | 1 | 3 | 1 | 1 | 3 | 1 | 1 | 2 | 2 | 1 | 2 | 2 | 2 | - |
| Sangiran 17 | 1 | 3 | 2 | 3 | 3 | 2 | 3 | 3 | 2 | 2 | 3 | 1 | 2 | 2 | 1 | 2 | 3 | 2 | 2 | 1 | 2 | 1 | 1 | 1 | 2 | 1 | 1 | 2 | 2 | 2 | 1 | 1 | 2 | 1 | 3 | 1 | 2 | 2 | 2 | 1 | 1 | 1 | - | 1 | 3 | 1 | 2 | 2 | 2 | 2 |
| **Ceprano** | **1** | **3** | **2** | **2** | **3** | **2** | **1** | **3** | **2** | **3** | **2** | **2** | **1** | **1** | **1** | **1** | **3** | **3** | **2** | **3** | **2** | **1** | **1** | **1** | **2** | **1** | **1** | **2** | **2** | **2** | **2** | **1** | **2** | **1** | **2** | **2** | **2** | **2** | **2** | **2** | **1** | **1** | **2** | **2** | **3** | **1** | **1** | **2** | **2** | **2** |
| SH5 | 3 | 3 | 2 | 2 | 3 | 3 | 2 | 3 | 2 | 2 | 2 | 2 | 2 | 1 | 2 | 2 | 2 | 3 | 1 | 3 | 2 | 2 | 1 | 2 | 1 | 3 | 2 | 2 | 2 | 2 | 2 | 2 | 2 | 1 | 3 | 2 | 2 | 3 | 2 | 2 | 1 | 1 | 1 | 1 | 3 | 1 | 1 | 2 | 1 | 2 |
| Steinheim | 2 | 2 | 2 | 2 | 3 | 3 | 1 | 3 | 3 | 3 | 1 | 1 | 1 | 1 | 2 | 2 | 2 | 3 | 1 | 2 | 1 | 2 | 2 | 2 | 1 | 3 | 2 | 1 | 3 | 2 | 1 | 2 | 2 | 1 | 2 | 2 | 2 | 3 | 1 | 2 | 1 | 1 | 1 | 1 | 3 | 1 | 2 | 2 | 1 | 2 |
| Petralona | 1 | 3 | 2 | 2 | 3 | 3 | 1 | 3 | 2 | 3 | 3 | 1 | 1 | 1 | 2 | 2 | 3 | 3 | 2 | 1 | 2 | 2 | 2 | 1 | 2 | 1 | 1 | 2 | 2 | 3 | 1 | 2 | 1 | 1 | 3 | 1 | 1 | 3 | 2 | 2 | 1 | 2 | 1 | 2 | 3 | 1 | 2 | 2 | 1 | 1 |
| Broken Hill 1 | 1 | 3 | 2 | 2 | 3 | 2 | 1 | 3 | 2 | 3 | 3 | 1 | 2 | 1 | 1 | 2 | 2 | 3 | 2 | 1 | 2 | 2 | 2 | 2 | 1 | 3 | 1 | 3 | 2 | 3 | 2 | 2 | 2 | 1 | 2 | 2 | 2 | 3 | 1 | 2 | 2 | 1 | 1 | 2 | 1 | 2 | 2 | 2 | 1 | 2 |
| Jebel Irhoud 1 | 3 | 1 | 1 | 1 | 3 | 3 | 3 | 3 | 3 | 3 | 2 | 1 | 1 | 1 | 1 | 2 | 2 | 2 | 1 | 3 | 1 | 2 | 1 | 2 | 1 | 3 | 1 | 1 | 1 | 1 | 1 | 1 | 1 | 1 | 2 | 1 | 2 | 3 | 1 | 2 | 1 | 1 | 1 | 2 | 1 | 2 | 2 | 1 | 1 | 2 |
| LH 18 | 3 | 2 | 2 | 1 | 3 | 2 | 1 | 2 | 3 | 2 | 2 | 1 | 1 | 1 | 2 | 2 | 2 | 3 | 1 | 3 | 1 | 2 | 1 | 2 | 1 | 3 | 1 | 1 | 1 | 1 | 1 | 1 | 2 | 1 | 1 | 1 | 1 | 1 | 1 | 1 | 2 | 1 | 1 | 2 | 2 | 2 | 1 | 1 | 2 | 1 |
| Omo II | 1 | 2 | 2 | 1 | 2 | 3 | 3 | 1 | 2 | 3 | 2 | 1 | 1 | 1 | 2 | 2 | 3 | 3 | 2 | 3 | 2 | 1 | 1 | 2 | 1 | 3 | 1 | 2 | 1 | 1 | 2 | 1 | 1 | 1 | 1 | 1 | 1 | 2 | 1 | 2 | 1 | 2 | 1 | 2 | 1 | 2 | 2 | 1 | 1 | 2 |
| Singa | 3 | 3 | 2 | 1 | 2 | 3 | 3 | 1 | 3 | 3 | 2 | 2 | 2 | 1 | 1 | 1 | 2 | 3 | 1 | 3 | 1 | 2 | 1 | 2 | 1 | 3 | 1 | 1 | 1 | 1 | 1 | 1 | 1 | 1 | 2 | 1 | 1 | 3 | 1 | 2 | 1 | 1 | 1 | 2 | 3 | 2 | 1 | 1 | 2 | 1 |
| ZH III | 1 | 3 | 2 | 2 | 3 | 2 | 2 | 3 | 3 | 2 | 2 | 1 | 2 | 2 | 2 | 2 | 2 | 1 | 2 | 1 | 2 | 1 | 1 | 1 | 2 | 2 | 1 | 3 | 2 | 2 | 1 | 1 | 2 | 2 | 3 | 2 | 2 | 3 | 1 | 1 | 2 | 2 | 2 | 2 | 3 | 3 | 1 | 2 | 2 | 2 |
| ZH XII | 1 | 3 | 2 | 2 | 3 | 2 | 2 | 3 | 2 | 2 | 3 | 1 | 2 | 2 | 2 | 2 | 3 | 3 | 2 | 1 | 2 | 1 | 1 | 1 | 1 | 1 | 1 | 3 | 2 | 2 | 1 | 1 | 2 | 2 | 3 | 2 | 2 | 2 | 1 | 2 | - | 1 | 2 | 2 | 3 | 3 | - | 2 | 1 | 2 |
| Dali | 3 | 3 | 2 | 2 | 3 | 3 | 1 | 3 | 2 | 3 | 3 | 1 | 2 | 1 | 1 | 2 | 3 | 3 | 2 | 3 | 2 | 1 | 1 | 1 | 2 | 1 | 1 | 1 | 2 | 2 | 2 | 2 | 1 | 1 | 3 | 2 | 2 | 3 | 2 | 1 | 1 | 1 | 1 | 2 | 3 | 2 | 2 | 2 | 2 | 2 |
| Jinniu Shan | 3 | 3 | 2 | 2 | 3 | 3 | 3 | 3 | 2 | 2 | 2 | 2 | 2 | 1 | 1 | 1 | 2 | 3 | 1 | 2 | 2 | 2 | 2 | 2 | 2 | 2 | 1 | 2 | 2 | 2 | 2 | 2 | 1 | 1 | 2 | 2 | 1 | 2 | 1 | 1 | 1 | 2 | 2 | 1 | 1 | 1 | 1 | 2 | 1 | 2 |
| Ngawi 1 | 1 | 3 | 1 | 3 | 3 | 3 | 2 | 2 | 2 | 2 | 2 | 1 | 2 | 2 | 2 | 2 | 2 | 3 | 2 | 2 | 2 | 2 | 1 | 1 | 1 | 2 | 1 | 2 | 2 | 3 | 1 | 1 | 2 | 2 | 2 | 2 | 2 | 3 | 1 | 2 | 1 | 1 | - | 2 | 2 | 3 | 1 | 1 | 2 | 1 |
| Ng 6 | 1 | 3 | 1 | 2 | 3 | 3 | 2 | 3 | 2 | 1 | 2 | 1 | 2 | 2 | 2 | 2 | 2 | 2 | 2 | 1 | 2 | 2 | 1 | 1 | 2 | 2 | 1 | 3 | 2 | 2 | 2 | 1 | 2 | 2 | 3 | 2 | 2 | 2 | 1 | 2 | 1 | 2 | 2 | 2 | 3 | 1 | 1 | 1 | 2 | 2 |
| Ng 14 | 1 | 3 | 1 | 2 | 3 | 2 | 2 | 3 | 2 | 2 | 2 | 1 | 2 | 2 | 2 | 2 | 2 | 3 | 2 | 1 | 2 | 2 | 1 | 1 | 2 | 3 | 1 | 3 | 2 | 3 | 2 | 1 | 2 | 2 | 3 | 2 | 2 | 2 | 1 | 2 | 1 | 2 | 2 | 2 | 3 | 3 | 1 | 1 | 2 | 1 |
| Gibraltar 1 | 2 | 1 | 2 | 2 | 3 | 3 | 3 | 3 | 3 | 1 | 2 | 2 | 1 | - | - | - | 2 | 3 | 1 | 1 | 2 | 2 | 2 | 2 | 1 | 2 | 3 | 1 | 3 | 2 | 1 | 1 | 1 | 1 | 3 | 2 | 2 | 2 | 1 | 1 | 3 | 1 | - | 1 | 2 | 1 | 1 | 2 | 1 | 2 |
| La Ferrassie 1 | 2 | 3 | 2 | 3 | 3 | 3 | 3 | 3 | 3 | 3 | 3 | 1 | 1 | 1 | 2 | 2 | 2 | 3 | 1 | 1 | 2 | 2 | 2 | 2 | 1 | 2 | 3 | 1 | 3 | 2 | 1 | 1 | 1 | 1 | 3 | 1 | 2 | 3 | 3 | 1 | 3 | 2 | 1 | 1 | 2 | 1 | 2 | 2 | 1 | 2 |
| La Quina H5 | 2 | 3 | 2 | 2 | 3 | 3 | 3 | 3 | 3 | - | 2 | 2 | 1 | 1 | 1 | 1 | 1 | 3 | 1 | 1 | 2 | 2 | 2 | 2 | 1 | 2 | 3 | 2 | 3 | 2 | 1 | 1 | 1 | 1 | 2 | 1 | 2 | 3 | 3 | 1 | - | 1 | - | 1 | 1 | 1 | 2 | 2 | 2 | 1 |
| Monte Circeo I | 2 | 3 | 2 | 3 | 3 | 3 | 3 | 3 | 3 | 2 | 2 | 2 | 1 | 1 | 2 | 1 | 3 | 3 | 1 | 1 | 2 | 2 | 2 | 2 | 1 | 2 | 3 | 2 | 3 | 2 | 1 | 2 | 2 | 1 | 3 | 1 | 1 | 3 | 3 | 1 | 3 | 1 | 1 | 1 | 2 | 1 | 2 | 1 | 1 | 1 |
| La Chapelle | 2 | 1 | 2 | 3 | 3 | 3 | 3 | 3 | 3 | 3 | 2 | 2 | 1 | 1 | 1 | 2 | 2 | 3 | 1 | 1 | 2 | 2 | 2 | 2 | 1 | 2 | 3 | 1 | 3 | 2 | 1 | 1 | 1 | 1 | 1 | 1 | 2 | 3 | 3 | 1 | 1 | 2 | 1 | 1 | 2 | 1 | 2 | 2 | 1 | 1 |
| Spy 1 | 2 | 1 | 2 | 3 | 3 | 3 | 3 | 3 | 3 | 2 | 2 | 2 | 1 | 1 | 2 | 2 | 3 | 3 | 1 | 1 | 2 | 2 | 2 | 2 | 1 | 2 | 3 | 1 | 3 | 2 | 1 | 1 | 1 | 1 | 3 | 2 | 2 | 2 | 3 | 1 | 3 | 1 | 1 | 2 | 2 | 1 | 2 | 1 | 2 | 1 |
| Cro-Magnon I | 3 | 1 | 2 | 1 | 2 | 3 | 3 | 1 | 3 | 3 | 2 | 1 | 1 | 1 | 1 | 2 | 3 | 2 | 1 | 3 | 1 | 1 | 1 | 2 | 2 | 3 | 1 | 1 | 1 | 1 | 2 | 2 | 2 | 1 | 3 | 2 | 1 | 3 | 1 | 2 | 1 | 1 | 1 | 2 | 2 | 2 | 2 | 1 | 2 | 1 |
| Abri Pataud 1 | 3 | 3 | 2 | 1 | 2 | 3 | 3 | 1 | 3 | 3 | 2 | 1 | 1 | 1 | 1 | 2 | 2 | 3 | 1 | 3 | 1 | 2 | 1 | 2 | 1 | 3 | 1 | 1 | 1 | 1 | 2 | 1 | 1 | 1 | 2 | 1 | 1 | 3 | 1 | 2 | 1 | 2 | 1 | 2 | 2 | 2 | 2 | 1 | 2 | 1 |
| Chancelade | 3 | 1 | 2 | 1 | 2 | 3 | 3 | 1 | 3 | 2 | 2 | 1 | 1 | 1 | 1 | 2 | 3 | 3 | 1 | 3 | 1 | 2 | 1 | 2 | 1 | 3 | 1 | 1 | 1 | 1 | 2 | 2 | 2 | 1 | 1 | 2 | 1 | 3 | 1 | 2 | 1 | 2 | 1 | 2 | 2 | 2 | 2 | 2 | 1 | 1 |
| Qafzeh 9 | 3 | 1 | 2 | 1 | 2 | 3 | 3 | 1 | 3 | 1 | 2 | 1 | 1 | 1 | - | 1 | 2 | 3 | 1 | 1 | 1 | 1 | 1 | 2 | 1 | 3 | 1 | 1 | 1 | 1 | 1 | 2 | 2 | 1 | 2 | 1 | 1 | 3 | 1 | 2 | - | 1 | - | 2 | 1 | 2 | 1 | 1 | 2 | 2 |
| Skhūl V | 3 | 1 | 1 | 2 | 3 | 2 | 3 | 3 | 3 | 2 | 2 | 1 | 1 | 1 | 2 | 2 | 2 | 3 | 1 | 3 | 2 | 2 | 1 | 2 | 2 | 3 | 1 | 1 | 1 | 1 | 2 | 2 | 1 | 1 | 1 | 2 | 1 | 3 | 1 | 2 | 1 | 1 | 1 | 2 | 1 | 2 | 2 | 1 | 2 | 2 |
| Ohalo II | 3 | 1 | 2 | 1 | 2 | 3 | 1 | 1 | 3 | 2 | 2 | 1 | 1 | 1 | 1 | 1 | 2 | 3 | 1 | 3 | 1 | 2 | 1 | 2 | 1 | 3 | 1 | 1 | 1 | 1 | 2 | 2 | 2 | 1 | 3 | 1 | 2 | 2 | 1 | 2 | 1 | 1 | 1 | 2 | 2 | 2 | 2 | 1 | 1 | 2 |
