# Supplementary material for: The Stem Species of Our Species: A Place for the Archaic Human Cranium from Ceprano, Italy
Source: PLoS One. 2011 Apr 20;6(4):e18821. doi: 10.1371/journal.pone.0018821 (PMC3080388; doi:10.1371/journal.pone.0018821)
Supplement: Table S13 — Hierarchical classification: general analysis (Figure S3A); classification consolidation through iterations. In successive iterations, the probability of the partition (i.e., the clustering process) is adjusted (i.e., individuals are randomly reallocated to the different clusters) to maximize the likelihood of the data given the number of clusters. The number of iterations is lower than 10 and the inertia does not vary. The partition is thus, solid. (DOC) [file pone.0018821.s016.doc]

**Table S13.**

| **Iteration** | **Total inertia** | **Inter-cluster inertia** | **Quotient** |
| --- | --- | --- | --- |
| 0 | 1.44000 | 0.55966 | 0.38865 |
| 1 | 1.44000 | 0.55966 | 0.38865 |
| 2 | 1.44000 | 0.55966 | 0.38865 |
