# Supplementary material for: The Stem Species of Our Species: A Place for the Archaic Human Cranium from Ceprano, Italy
Source: PLoS One. 2011 Apr 20;6(4):e18821. doi: 10.1371/journal.pone.0018821 (PMC3080388; doi:10.1371/journal.pone.0018821)
Supplement: Table S15 — Hierarchical classification, general analysis (Figure S3A): description of modern Humans and Neandertals clusters by the most relevant morphological features and character states. The statistical analysis identifies the character states that contribute the most to the formation of each class. The T-Value (pertinence criterion) must be ≥2 at p<0.05. (DOC) [file pone.0018821.s018.doc]

**Table S15.**

| **Modern humans** | | | | **Neandertals** | | | |
| --- | --- | --- | --- | --- | --- | --- | --- |
| **Morphological features** | **character states** | **T-Values** | **p** | **Morphological features** | **character states** | **T-Values** | **p** |
| *Torus occipitalis transversus* | 1 | 8.84 | 5E-19 | Position of the auditory meatus | 3 | 4.97 | 3E-07 |
| *Torus occipitalis transversus* form in *norma occipitalis* | 1 | 8.84 | 5E-19 | *Suprainiac fossa* | 3 | 4.97 | 3E-07 |
| Supra-orbital region: *sulcus supraorbitalis* | 1 | 8.51 | 9E-18 | *Torus occipitalis transversus* | 3 | 4.65 | 2E-06 |
| Sharply angulated occipital. *norma lateralis* | 1 | 7.49 | 4E-14 | Supra-orbital region: *sulcus supraorbitalis* | 3 | 4.42 | 5E-06 |
| Sulcus postorbitalis | 1 | 7.28 | 2E-13 | Outline of the calvaria. *norma lateralis* | 2 | 4.42 | 5E-06 |
| *Tuber parietale* | 3 | 6.83 | 4E-12 | Juxtamastoid ridge development / *processus mastoidus* | 3 | 4.01 | 3E-05 |
| Outline of the calvaria. *norma lateralis* | 3 | 6.36 | 1E-10 | Occipital bun | 2 | 3.94 | 4E-05 |
| Articular tubercle configuration | 2 | 6.08 | 6E-10 | Outline of the *planum occipitalis*. *norma occipitalis* | 2 | 3.59 | 2E-04 |
| *Tuberculum supramastoideum anterius* | 1 | 5.99 | 1E-09 | *Tuber parietale* | 1 | 3.59 | 2E-04 |
| *Tuber frontale* | 3 | 5.88 | 2E-09 | *Processus mastoidus*: downward development / basicranium | 1 | 3.59 | 2E-04 |
| *Sulcus supratoralis* | 1 | 5.73 | 5E-09 | *Torus occipitalis transversus* form in *norma occipitalis* | 2 | 3.50 | 2E-04 |
| Outline of the *planum occipitalis*. *norma occipitalis* | 3 | 5.53 | 3E-08 | Sharply angulated occipital. *norma lateralis* | 2 | 3.02 | 0.001 |
| Petro-tympanic crest orientation | 2 | 5.35 | 4E-08 | *Sulcus postorbitalis* | 3 | 3.02 | 0.001 |
| Position of the auditory meatus | 1 | 5.05 | 2E-07 | Projection of the supra-orbital region | 3 | 2.95 | 0.002 |
| Opisthocranion coincident with inion | 2 | 5.05 | 2E-07 | Articular tubercle configuration | 1 | 2.88 | 0.002 |
| Development of the *crista supramastoidea* at the porion | 1 | 4.96 | 4E-07 | Medio-sagittal supra-glabellar tubercle | 2 | 2.60 | 0.005 |
| Outline of the supra-orbital region. *norma verticalis* | 3 | 4.68 | 1E-06 | Development of the *crista supramastoidea* at the porion | 3 | 2.51 | 0.006 |
| *Torus angularis parietalis* | 1 | 4.64 | 2E-06 | *Protuberantia occipitalis externa* | 1 | 2.47 | 0.007 |
| Sagittal keel on the frontal | 1 | 4.64 | 2E-06 | *Tuberculum supramastoideum anterius* | 2 | 2.33 | 0.010 |
| Occipital bun | 1 | 4.64 | 2E-06 | - | - | - | - |
| *Processus mastoidus*: downward development / basicranium | 2 | 4.47 | 4E-06 | - | - | - | - |
| Juxtamastoid ridge development / *processus mastoidus* | 1 | 4.19 | 1E-05 | - | - | - | - |
| *Crista supramastoidea* continues with the *processus zygomaticus temporalis* | 1 | 4.19 | 1E-05 | - | - | - | - |
| *Suprainiac fossa* | 1 | 4.17 | 2E-05 | - | - | - | - |
| Projection of the supra-orbital region | 2 | 4.17 | 2E-05 | - | - | - | - |
| Sagittal keel on the bregma-lambda arc | 1 | 4.07 | 2E-05 | - | - | - | - |
| *Processus retromastoideus* | 1 | 4.04 | 3E-05 | - | - | - | - |
| Bregmatic eminence | 1 | 3.69 | 1E-4 | - | - | - | - |
| *Crista occipitomastoidea* | 1 | 3.69 | 1E-4 | - | - | - | - |
| Parasagittal hollowing on both sides of the parietal suture | 1 | 3.58 | 2E-4 | - | - | - | - |
| Projection of the supra-orbital region | 1 | 3.48 | 3E-4 | - | - | - | - |
| Outline of the superior border of the squama | 1 | 3.24 | 6E-4 | - | - | - | - |
| Frontal cord length / parietal cord length | 1 | 2.96 | 0.001 | - | - | - | - |
| Postorbital constriction | 3 | 2.93 | 0.001 | - | - | - | - |
| Glenoid cavity depth | 2 | 2.86 | 0.002 | - | - | - | - |
| Temporal squama height | 2 | 2.70 | 0.003 | - | - | - | - |
| Medio-sagittal supra-glabellar tubercle | 1 | 2.66 | 0.004 | - | - | - | - |
| *Protuberantia occipitalis externa* | 2 | 2.52 | 0.006 | - | - | - | - |
| Supramastoid groove | 3 | 2.40 | 0.008 | - | - | - | - |
| Antero-posterior convexity of the frontal | 3 | 2.40 | 0.008 | - | - | - | - |
